# Follistatin promotes LIN28B-mediated supporting cell reprogramming and hair cell regeneration in the murine cochlea

**DOI:** 10.1126/sciadv.abj7651

**Published:** 2022-02-11

**Authors:** Xiao-Jun Li, Charles Morgan, Loyal A. Goff, Angelika Doetzlhofer

**Affiliations:** 1 The Solomon H. Snyder Department of Neuroscience, Johns Hopkins University School of Medicine, Baltimore, MD 21205, USA.; 2 McKusick-Nathans Institute for Genetic Medicine, Johns Hopkins University School of Medicine, Baltimore, MD 21205, USA.; 3 Kavli Neuroscience Discovery Institute, Johns Hopkins University, Baltimore, MD 21205, USA.; 4 Department of Otolaryngology and Center for Hearing and Balance, Johns Hopkins University School of Medicine, Baltimore, MD 21205, USA.

## Abstract

Hair cell (HC) loss within the inner ear cochlea is a leading cause for deafness in humans. Before the onset of hearing, immature supporting cells (SCs) in neonatal mice have some limited capacity for HC regeneration. Here, we show that in organoid culture, transient activation of the progenitor-specific RNA binding protein LIN28B and Activin antagonist follistatin (FST) enhances regenerative competence of maturing/mature cochlear SCs by reprogramming them into progenitor-like cells. Transcriptome profiling and mechanistic studies reveal that LIN28B drives SC reprogramming, while FST is required to counterbalance hyperactivation of transforming growth factor–β–type signaling by LIN28B. Last, we show that LIN28B and FST coactivation enhances spontaneous cochlear HC regeneration in neonatal mice and that LIN28B may be part of an endogenous repair mechanism that primes SCs for HC regeneration. These findings indicate that SC dedifferentiation is critical for HC regeneration and identify LIN28B and FST as main regulators.

## Teaser

Transient induction of LIN28B and FST enhances the regenerative plasticity of cochlear supporting cells in mice.

## INTRODUCTION

Proper function of the auditory sensory organ is essential for our ability to hear. Dysfunction or the loss of its mechanoreceptors, termed hair cells (HCs) due to genetic or environmental causes, is permanent and is a leading cause of hearing impairments and deafness in humans. Nonmammalian vertebrate species, such as birds and fish, regenerate HCs through mitotic and nonmitotic mechanisms [reviewed in (*1*)]. New HCs originate from neighboring glia-like supporting cells (SCs), with which HCs have a close lineage relationship. While cochlear SCs in adult mammals fail to regenerate HCs, recent studies revealed that cochlear SCs in perinatal mice have some limited capacity for HC regeneration (*2*, *3*). HC regeneration engages many of the same gene programs that have been previously found to be critical for the generation of HCs during development. Cochlear HCs and SCs originate from a common pool of progenitor cells, termed prosensory cells. Specification and proliferation of cochlear prosensory cells depend on the complex interplay of Notch, fibroblast growth factor receptor 1 (FGFR1), bone morphogenetic protein (BMP), and Wnt/β-catenin signaling [reviewed in (*4*)]. Following terminal mitosis, prosensory cells fated to differentiate into HCs up-regulate ATOH1, a basic helix-loop-helix transcription factor essential for HC fate specification and differentiation (*5*). The remaining prosensory cells acquire a SC fate, a choice reinforced by Notch signaling (*6*). Consistent with their developmental roles, overactivation of Wnt/β-catenin signaling (*7*, *8*) or ectopic activation of *Atoh1* expression stimulates cochlear SCs in neonatal mice/tissue to reenter the cell cycle and to form new HCs (*9*, *10*). Moreover, inhibition of Notch signaling induces direct conversion of SCs into HCs (transdifferentiation) (*11*, *12*). However, cochlear HCs and SCs in mice are not functional (mature) until the onset of hearing at postnatal day 12 (P12) (*13*), and little to no HC production is observed in response to ectopic expression of *Atoh1* (*9*, *10*), Wnt activation (*14*), and/or Notch inhibition (*15*, *16*) starting at P5.

The failure of later stage SCs to respond to HC fate–inducing cues has been recently linked to diminished LIN28B expression and rising *let-7* microRNA (miRNA) expression (*16*, *17*). LIN28B and its paralog LIN28A are RNA binding proteins that promote stemness, cell proliferation, and reprogramming through enhancing transcript stability and translation of pro-growth mRNA targets, as well as through blocking the biogenesis of the growth-inhibitory *let-7* family of miRNAs. In turn, *let-7* miRNAs bind to the 3′ untranslated region of *Lin28a* and *Lin28b* mRNAs and prevent their translation [reviewed in (*18*)]. We recently showed that immature cochlear SCs that overexpress *let-7* miRNAs, or that are deficient for *Lin28b* and *Lin28a*, fail to produce HCs in response to HC fate–inducing cues. Moreover, we found that overexpression of human *LIN28B* enables stage P5 cochlear SCs to form new HCs in response to regenerative cues and showed that such LIN28B-enabled HC production requires the activation of mammalian target of rapamycin (mTOR) signaling (*16*). In addition, our recent study uncovered that *LIN28B* activation up-regulates the expression of follistatin (*Fst*). FST is a secreted protein that functions as an antagonist of Activin-type ligands, which are members of the transforming growth factor–β (TGF-β) superfamily (*19*). In the developing murine cochlea, *Fst* is highly expressed in prosensory cells and functions in keeping prosensory cells in a proliferative, undifferentiated state through antagonizing Activin A–mediated signaling (*20*). However, the role of FST in HC regeneration remains to be addressed.

Here, we establish FST as an essential cofactor for LIN28B-mediated SC reprogramming and HC regeneration in the murine cochlea. We show that coactivation of FST and LIN28B enables cochlear SCs, isolated from hearing juvenile mice, to proliferate and form new HCs in vitro. Gene expression and mechanistic studies reveal that LIN28B is the main driver of the reprogramming of SCs into progenitor-like cells, while FST is required to counterbalance aberrant activation of TGF-β signaling by LIN28B. In particular, we show that *Fst* knockdown diminishes LIN28B’s ability to promote HC production, while knockdown of the TGF-β ligand *Tgfb2* enhances it. Last, we provide evidence that *Lin28a* and *Lin28b* induction and suppression of TGF-β signaling are part of an endogenous repair mechanism that supports spontaneous HC regeneration in neonatal mice.

## RESULTS

### Coactivation of FST and LIN28B boosts the HC-forming capacity of cochlear SCs

To address whether FST promotes SC plasticity, we transiently overexpressed FST by itself or in combination with LIN28B in stage P5 cochlear tissue/cells and exposed the tissue/cells to mitotic and HC fate–inducing signals. To overexpress FST and/or LIN28B, we made use of doxycycline (dox)–inducible *iFST* and *iLIN28B* transgenic (tg) mouse models, which allow for robust overexpression of human *FST* and human *LIN28B* in cochlear cells/tissue (*16*, *17*, *20*).

First, we analyzed whether reactivation of FST by itself or in combination with LIN28B enables stage P5 SCs to convert into HCs in response to Wnt activation and Notch inhibition. Recent studies found that combined Wnt activation and Notch inhibition is highly effective in stimulating HC formation in stage P0 to P2 cochlear explants (*14*). However, at later stages (P4/P5 and later), such treatment is ineffective, but responsiveness to these HC fate–inducing cues can be restored by LIN28B reactivation (*16*). We isolated cochlear sensory epithelia including its surrounding mesenchymal and neuronal tissue from five-day-old control, *iFST*, *iLIN28B*, and *iFST;iLIN28B* tg mice and cultured these “cochlear explants” in the presence of the glycogen synthase kinase 3β inhibitor CHIR99021 (activates Wnt signaling), the γ-secretase inhibitor LY411575 (blocks Notch signaling), and dox for 4 days (fig. S1A). To detect new HCs, we stained cochlear explants for myosin VIIa (MYO7A), a HC-specific protein, and the transcription factor SOX2. Cochlear HCs at stages P5 and later lack SOX2 expression, while newly formed HCs highly express SOX2, which allows one to distinguish between new HCs (MYO7A^+^SOX2^+^) and preexisting HCs (MYO7A^+^ only) (*21*). As anticipated, we found that control cochlear explants produced only few new HCs (MYO7A^+^SOX2^+^) within the cochlear apex, while LIN28B (*iLIN28B*) overexpression substantially increased HC formation in the cochlear apex (~fourfold) compared to control and broadened the region that formed new HCs to include the midportion of the cochlea. FST (*iFST*) overexpression had a similar positive effect on HC formation, yielding close to fourfold more new HCs compared to control, an effect that was further enhanced by FST and LIN28B coactivation (*iFST;iLIN28B*), which further extended the region that produced new HCs to the basal portion of cochlear explants (fig. S1, B and C). Combined activation of FST and LIN28B resulted also in a mild increase in the number of HCs that incorporated the thymidine analog EdU (5-ethynyl-2′-deoxyuridine), compared to LIN28B only (fig. S1, B and D). However, the vast majority of newly formed HCs (~90%) lacked EdU labeling. Collectively, these results indicate that FST enhances, similar to LIN28B, the capacity of SCs to transdifferentiate into HCs in response to HC fate–inducing cues, an effect that is further enhanced by coactivation of FST and LIN28B.

**Fig. 1.**
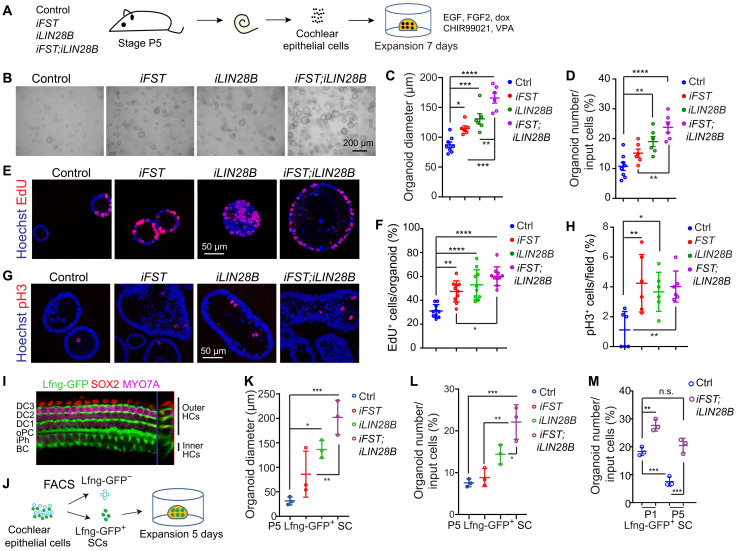
Coactivation of FST and LIN28B greatly enhances the progenitor potential of stage P5 cochlear SCs. Cochlear epithelial cells (B to H) or FACS-purified Lfng-GFP^+^ cochlear SCs (K to L) from P5 control, *iFST*, *iLIN28B*, and *iFST*;*iLIN28B* tg mice were used to establish organoid cultures. (**A**) Experimental scheme (B to H). (**B**) Low-power bright-field (BF) images of organoid cultures. (**C**) Organoid diameters in (B) (*n* = 8, control; *n* = 6, other groups; three independent experiments). (**D**) Organoid-forming efficiency in (B) (*n* = 8, control; *n* = 6, other groups; three independent experiments). (**E**) Organoids stained for EdU (red) incorporations. (**F**) Percentage of EdU^+^ cells per organoid in (E) (*n* = 10, three independent experiments). (**G**) Organoids immunostained for phospho–histone 3 (pH3). (**H**) Percentage of pH3^+^ cells per field in (G) (*n* = 6, two independent experiments). (**I**) *Lfng-GFP* transgene expression in P5 cochlear tissue. MYO7A (magenta) marks HCs. SOX2 (red) and Lfng-GFP (green) labels SCs. DC1-3, Deiter cells; 1-3; oPC, outer pillar cell; iPh, inner phalangeal cell; BC, border cell. (**J**) Experimental scheme (K to M). (**K** and **L**) Organoid diameter (K) and organoid formation efficiency (L) (*n* = 3, two independent experiments). (**M**) Organoid-forming efficiency of control and FST + LIN28B–overexpressing SCs, stages P1 and P5 (*n* = 3, two independent experiments). One-way analysis of variance (ANOVA) (C to L) or two-way ANOVA (M) with Tukey’s correction was used to calculate *P* values. *P < 0.05, **P < 0.01, ***P < 0.001, and ****P < 0.0001. n.s., not significant.

We next analyzed whether FST by itself or in combination with LIN28B enhances the capacity of stage P5 cochlear epithelial cells (SCs) to form HCs through a mitotic mechanism using a modified version of a recently developed organoid culture platform (*22*). To enable the formation of organoids, we cultured dissociated cochlear epithelial cells (includes SCs) at high density in a drop of Matrigel matrix in an expansion medium containing epidermal growth factor (EGF) and fibroblast growth factor 2 (FGF2), as well as the Wnt agonist CHIR99021 and histone deacetylase inhibitor valproic acid (VPA) (Fig. 1A). We found that similar to LIN28B, FST-overexpressing cultures (*iFST*) formed more organoids (1.5-fold) that were, on average, twofold larger than control, an effect that was further enhanced by combined FST and LIN28B overexpression (*iFST;iLIN28B*) (Fig. 1, B to D). The observed increase in the number and size of FST- and/or LIN28B-overexpressing organoids was, in large part, due to an increase in cell proliferation. Two-hour EdU pulse experiments revealed that the percentage of cells in S phase (percentage of EdU^+^ cells) was about 1.5-fold higher in FST- or LIN28B-overexpressing organoids compared to control organoids and twofold higher in FST + LIN28B–overexpressing organoids (Fig. 1, E and F). Moreover, immunostaining against phospho–histone 3 (pH3) revealed that the percentage of cells undergoing mitosis was about threefold higher in FST-, LIN28B-, and FST + LIN28B–overexpressing organoids than in control (Fig. 1, G and H).

We obtained qualitative similar results when we used fluorescence-activated cell sorting (FACS)–purified cochlear SCs from 5-day-old control, *iFST*, *iLIN28B*, and *iFST;iLIN28B* tg mice as starting material. To enable FACS purification of cochlear SCs, we used Lfng–green fluorescent protein (GFP) tg mice, which express GFP in the majority of cochlear SC subtypes including Deiters’ cells, outer pillar cells, inner phalangeal cells, and border cells (Fig. 1, I and J, and fig. S2G) (*11*). We found that SCs that coexpressed FST and LIN28B formed 1.5 times more organoids than LIN28B-overexpressing SCs and formed three times more organoids than control SCs, which, on average, were 1.5 times larger than LIN28B-overexpressing organoids and eight times larger than control organoids (Fig. 1, K and L). To address whether combined overexpression of FST and LIN28B in stage P5 SCs restores organoid formation efficiency to a level similar to that observed at neonatal stages, we established organoid cultures with stage P1 and stage P5 control and FST + LIN28B–overexpressing SCs. As anticipated, we found that stage P1 control SCs had a significantly higher organoid formation efficiency (percentage of plated SCs that form organoids) than their stage P5 counterparts (Fig. 1M). Moreover, we found that the colony-forming efficiency (~20%) of stage P5 FST + LIN28B–overexpressing SCs was similar to that of stage P1 control SCs (Fig. 1M).

**Fig. 2.**
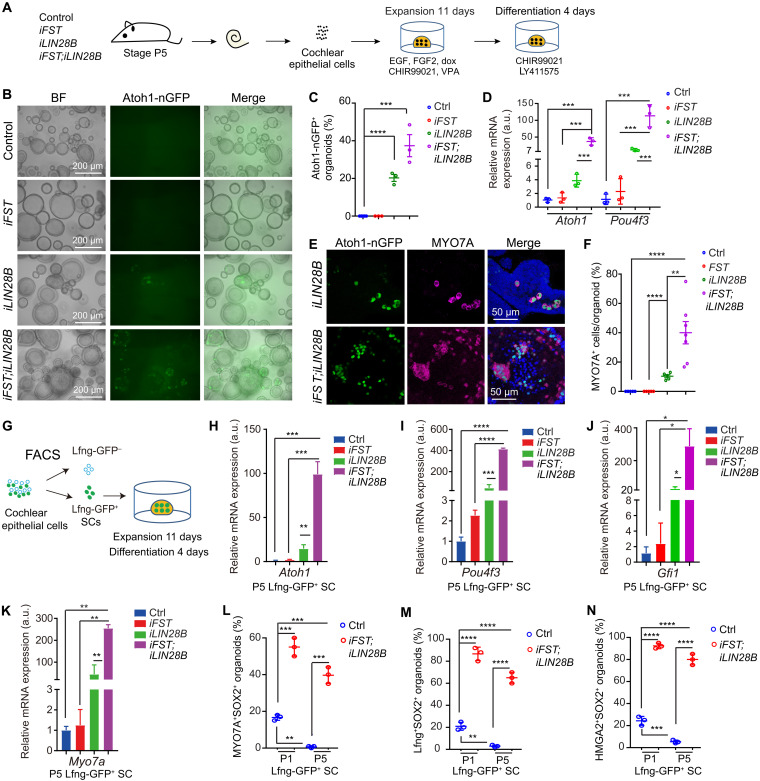
Coactivation of FST and LIN28B greatly enhances the HC-forming capacity of stage P5 cochlear SCs. Cochlear epithelial cells (B to F) or FACS-purified cochlear SCs (G to N) from P5 control, *iFST*, *iLIN28B*, and *iFST*;*iLIN28B* tg mice were used to establish organoid cultures. Cultures were analyzed after 4 days of differentiation. (**A**) Experimental scheme (B to F). Atoh1-nGFP marks nascent HCs. (**B**) Low-power bright-field and green fluorescent (Atoh1-nGFP) images of organoid cultures. (**C**) Percentage of Atoh1-nGFP^+^ organoids in (B) (*n* = 5, control; *n* = 3, all other groups; two independent experiments). (**D**) Reverse transcription polymerase chain reaction (RT-PCR) of HC-specific (*Atoh1* and *Pou4f3*) mRNA expression in organoids (*n* = 3, from one representative experiment and two independent experiments). a.u., arbitrary units. (**E**) Atoh1-GFP^+^ (green) organoids immunostained for HC-specific protein MYO7A (magenta). (**F**) Percentage of MYO7A^+^ HCs per organoid in (E) (*n* = 7, *iFST*;*iLIN28B*; *n* = 5, other groups; two independent experiments). (**G**) Experimental scheme (G to N). (**H** to **K**) RT-PCR of HC-specific *Atoh1* (H), *Pou4f3* (I), *Gfi1* (J), and *Myo7a* (K) mRNA expression (*n* = 3). (**L** to **N**) MYO7A, SOX2, and HMGA2 immunostaining and Lfng-GFP reporter expression was used to analyze the percentage of organoids that contained HCs (MYO7A^+^SOX2^+^) (L), Lfng^+^SOX2^+^ cells (M), and HMGA2^+^SOX2^+^ cells (N) in SC-derived control and FST + LIN28B–overexpressing cultures stages P1 and P5 (*n* = 3, two independent experiments). One-way ANOVA (C to K) or two-way ANOVA (L to N) with Tukey’s correction was used to calculate *P* values. *P < 0.05, **P < 0.01, ***P < 0.001, and ****P < 0.0001.

We next analyzed whether FST overexpression by itself or in combination with LIN28B enhances HC production. Newly formed HCs were identified by Atoh1-nuclear GFP (nGFP) reporter expression (*23*). To stimulate HC differentiation, we switched the dox-containing expansion medium to a differentiation medium containing Wnt agonist CHIR99021 and Notch inhibitor LY411575 (Fig. 2A). We found that after four days of differentiation, *iFST* and control cultures failed to produce Atoh1-nGFP^+^ organoids, while 20% of organoids in *iLIN28B* cultures and close to 40% of organoids in *iFST;iLIN28B* cultures contained Atoh1-nGFP^+^ cell clusters (Fig. 2, B and C). Consistent with the twofold increase in Atoh1-nGFP^+^ organoids, HC-specific transcripts (*Atoh1* and *Pou4f3*) were increased by 10-fold in *iFST;iLIN28B* cultures compared with *iLIN28B* cultures (Fig. 2D). Furthermore, we found that in *iFST;iLIN28B* cultures, the percentage of MYO7A^+^ HCs was fourfold higher than in *iLIN28B* cultures, and we detected little to no MYO7A^+^ HCs in control or *iFST* cultures (Fig. 2, E and F).

Similar results were obtained when we used FACS-purified cochlear Lfng-GFP^+^ SCs from stage P5 mice as starting material (Fig. 2G and fig. S2G). We found that coactivation of FST and LIN28B significantly boosted HC formation compared to LIN28B alone, with *iFST;iLIN28B* organoids expressing HC fate–inducing transcription factors (*Atoh1*, *Pou4f3*, and *Gfi1*) and the HC marker gene *Myo7a* at a three- to fivefold higher level than *iLIN28B* organoids (Fig. 2, H to K). The capacity of SCs to form HCs correlated with their ability to transiently down-regulate Lfng-GFP expression during expansion. We found that after 11 days of expansion, only 6% of organoids in *iFST;iLIN28B* cultures and 16% of organoids in *iLIN28B* cultures contained cells that highly expressed Lfng-GFP, suggesting that the majority of SCs in these cultures may have dedifferentiated into progenitor-like cells. By contrast, 51% of organoids in control and 39% of organoids in *iFST* cultures contained cells that highly expressed Lfng-GFP (fig. S2, A to D). We next tested whether overexpression of FST + LIN28B in stage P5 cochlear SCs boosts the capacity for HC formation to a level similar to that observed at neonatal stages. To do so, we established organoid cultures with control and *iFST;iLIN28B* tg Lfng-GFP^+^ SCs, stages P1 and P5, and after 4 days of differentiation stained for MYO7A, SOX2, and the progenitor marker HMGA2 (fig. S2, E and F). The architectural transcription factor HMGA2 is a *let-7* target and is essential for self-renewal of neural stem cell in the peripheral and central nervous system (*24*, *25*). In the murine cochlea, *Hmga2* is highly expressed in prosensory cells, and in differentiating SCs, but is close to undetectable in SCs and HCs after the onset of maturation (*17*, *26*). Our analysis revealed that the percentage of organoids that contained clusters of newly formed HCs (MYO7A^+^SOX2^+^) was two times higher in cultures that were established with P5 FST + LIN28B–overexpressing SCs than P1 control SCs (Fig. 2L). Consistent with the higher rate of HC formation, the percentage of organoids that contained clusters of Lfng-GFP^+^SOX2^+^ cells, representing SCs (Lfng-GFP^+^SOX2^+^MYO7A^−^) and inner HCs-like cells (Lfng-GFP^+^SOX2^+^MYO7A^+^) (*27*) (fig. S2F), was three times higher in cultures established with stage P5 FST + LIN28B–overexpressing SCs than P1 control SCs (Fig. 2M). Furthermore, the percentage of organoids that contained clusters of HMGA2^+^SOX2^+^ cells, representing progenitor-like cells (fig. S2E), was four times higher in cultures established with P5 FST + LIN28B–overexpressing SCs than P1 control SCs (Fig. 2N). Collectively, these results indicate that while FST overexpression by itself fails to enhance the HC-forming capacity of stage P5 cochlear SCs, its coactivation with LIN28B greatly enhances LIN28B’s positive effect on HC formation and boosts the HC-forming capacity of stage P5 cochlear SCs to a level that is significantly higher than that observed for stage P1 control cochlear SCs.

**Fig. 3.**
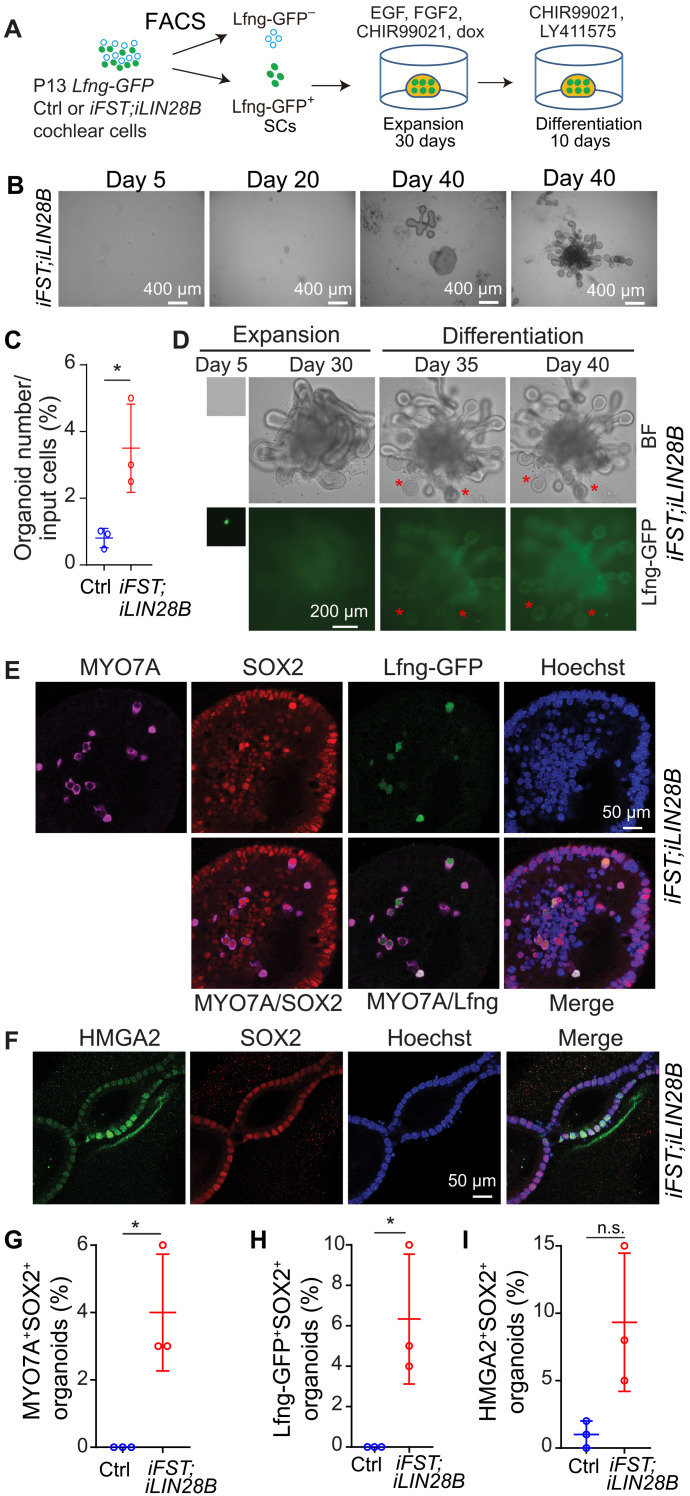
Coactivation of FST and LIN28B enables stage P13 cochlear SCs to form HCs. (**A**) Experimental scheme. Organoid cultures were established with FACS-purified Lfng-GFP^+^ cochlear SCs from stage P13 control and *iFST;iLIN28B* tg mice. (**B**) Low-power bright-field images of *iFST;iLIN28B* tg organoid culture. (**C**) Organoid formation efficiency of control (Ctrl) and FST + LIN28B (*iFST;iLIN28B*)–overexpressing SCs analyzed at 30 days of expansion (*n* = 3, two independent experiments). (**D**) Bright-field and green fluorescent (Lfng-GFP) images of *iFST;LIN28B* organoid culture. Red asterisks label organoids that induced Lfng-GFP expression after 5 days of differentiation (day 35). (**E** to **I**) Control and *iFST;iLIN28B* tg organoids were harvested after 10 days of differentiation and screened for Lfng-GFP expression. Lfng-GFP^+^ organoids were immunostained for MYO7A (magenta) and SOX2 (red) to identify new HCs. Lfng-GFP^−^ organoids were immunostained for progenitor cell marker HMGA2 (green) and SOX2 (red) to identify progenitor-like cells. Hoechst staining (blue) marks cell nuclei. (E) High-power images of HC cluster (MYO7A^+^SOX2^+^) in *iFST;iLIN28B* tg organoid. Note that some HCs coexpressed Lfng-GFP (green). (F) High-power images of SOX2^+^HMGA2^+^ cell cluster in *iFST;LIN28B* tg organoid. (G to I) Percentage of MYO7A^+^SOX2^+^ (G), Lfng-GFP^+^SOX2^+^ (H), and HMGA2^+^SOX2^+^ (I) organoids (*n* = 3, two independent experiments). Two-tailed, unpaired *t* test was used to calculate *P* values. *P < 0.05.

At about 12 days old (P12), mice start to hear, and cochlear HCs and SCs are considered functional mature (*13*). To determine whether transient activation of *FST* and *LIN28B* would enable functional mature cochlear SCs to reenter the cell cycle and form HC-containing organoids, we established organoid culture with FACS-purified Lfng-GFP^+^ SCs isolated from stage P13 *iFST;iLIN28B* tg mice and their control littermates (Fig. 3A and fig. S2H). Our analysis revealed that the organoid formation efficiency (percentage of plated SCs that form organoids) of P13 FST + LIN28B–overexpressing SCs was approximately 4%, which was five times lower than that observed for P1 control SCs or stage P5 FST + LIN28B–overexpressing SCs (Fig. 3, B and C). While control organoids failed to expand, FST + LIN28B–overexpressing organoids slowly increased in size and down-regulated Lfng-GFP expression during the 30-day expansion phase (Fig. 3D). Upon differentiation, about 5 to 10% of organoids reactivated Lfng-GFP expression, and after 10 days of differentiation, organoids were harvested and immunostained for MYO7A, SOX2, and HMGA2 and SOX2 to detect newly differentiated HCs (MYO7A^+^SOX2^+^), SCs (Lfng-GFP^+^SOX2^+^MYO7A^−^) (Fig. 3E), and progenitor-like-cells (HMGA2^+^SOX2^+^) (Fig. 3F). Our analysis revealed that about 4% of organoids contained newly formed HCs (MYO7A^+^SOX2^+^) (Fig. 3G) and 6% of organoids contained SCs (Lfng-GFP^+^SOX2^+^MYO7A^−^) and inner HC–like cells (Lfng-GFP^+^SOX2^+^MYO7A^+^) (Fig. 3H). Furthermore, we found that 10% of organoids contained clusters of progenitor-like cells (HMGA2^+^SOX2^+^) (Fig. 3, F and I). In summary, our data indicate that combined FST and LIN28B overexpression partially restores the regenerative competence of stage P13 cochlear SCs, with organoid formation efficiency and HC formation efficiency being about one-fifth of the rate observed with stage P1 cochlear control SCs.

### FST and LIN28B reprogram SCs into progenitor-like cells primed for HC fate induction

**Fig. 4.**
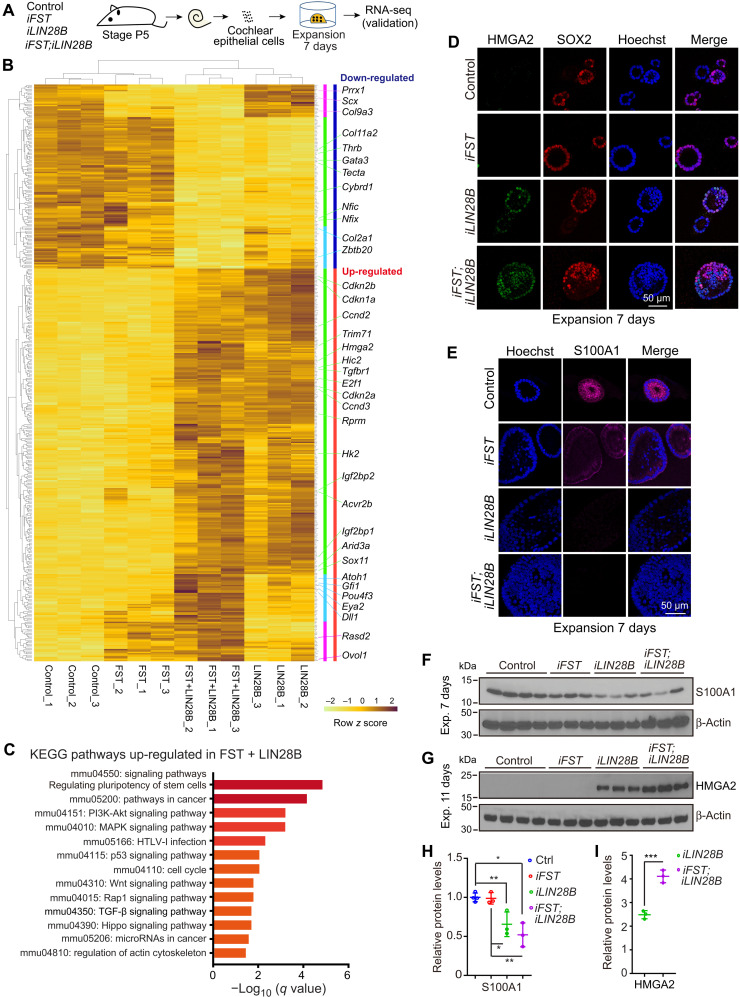
Coactivation of FST and LIN28B reprograms stage P5 cochlear SCs into HC progenitor-like cells. (**A**) Experimental scheme. Organoid cultures were established with cochlear epithelial cells from stage P5 control, *iFST*, *iLIN28B*, and *iFST*;*iLIN28B* tg mice. After 7 days of expansion, gene expression in control, LIN28B, FST, and FST + LIN28B organoids was analyzed using RNA sequencing (RNA-seq). (**B**) Heatmap of row *z* scores computed for DEGs between LIN28B + FST and control condition. Row *z* scores ranging from brown to yellow (up- to down-regulated). Red-colored vertical bar marks genes up-regulated, and blue-colored vertical bar marks genes down-regulated compared to control. Magenta-colored bars mark FST-regulated genes, green-colored bars mark LIN28B-regulated genes, and cyan-colored bars mark FST + LIN28B–regulated genes. (**C**) Kyoto Encyclopedia of Genes and Genomes (KEGG) pathways associated with LIN28B + FST up-regulated genes ranked by adjusted *P* value (*q* value). (**D**) High-power images of organoids immunostained for SOX2 (red) and progenitor marker HMGA2 (green). (**E**) High-power images of organoids immunostained for SC marker S100A1. (**F**) Immunoblots of S100A1 and β-actin proteins in organoids. (**G**) Immunoblots of HMGA2 and β-actin protein in organoids. (**H**) Quantification of S100A1 protein in (F) (*n* = 4, control; *n* = 3, other groups; from one representative experiment and two independent experiments). (**I**) Quantification of HMGA2 protein in (G) (*n* = 4, control; *n* = 3, other groups; from one representative experiment and two independent experiments). One-way ANOVA with Tukey’s correction was used to calculate *P* values. *P < 0.05, **P < 0.01, and ***P < 0.001.

To identify how FST and LIN28B coactivation boosts regenerative competence, we analyzed the transcriptome of P5 control, FST-, LIN28B-, and FST + LIN28B–overexpressing organoids after 7 days of expansion using RNA sequencing (Fig. 4A). The seven-day time point was chosen as it correlates with the peak of SC proliferation and presumed SC reprogramming (dedifferentiation into progenitor-like cells) (*16*). We used kallisto (v0.46.1) (*28*) to pseudo-align reads to the reference mouse transcriptome (Ensembl *Mus musculus* v96) and to quantify transcript abundance. The companion tool sleuth was used to determine differentially expressed genes (DEGs) comparing control to LIN28B-and/or FST-overexpressing conditions (*29*). Applying Wald tests, we identified 423 DEGs for FST + LIN28B (*q* < 0.01) (table S1), 764 DEGs for LIN28B (*q* < 0.01) (table S2), and 37 DEGs for FST (*q* < 0.01) (table S3) with control functioning as baseline. Unsupervised hierarchical clustering grouped FST + LIN28B DEGs into LIN28B-regulated (green), FST-regulated (magenta), and FST + LIN28B–coregulated (cyan) gene clusters (Fig. 4B). The list of down-regulated genes included SC-specific genes involved in tectorial membrane and extracellular matrix formation [*Tecta* (*30*), *Col2a1*, *Col11a2*, and *Col9a3*], as well as transcription factors known to promote glial cell differentiation [*Zbtb20* (*31*) and *Nfix* and *Nfic* (*32*)] and cochlear maturation (*Thrb*) (*33*). The list of up-regulated genes was dominated by LIN28B-regulated genes, including *let-7* target genes that promote stemness [e.g., *Hmga2* (*25*) and *Trim71* (*34*)], as well as genes involved in cell cycle regulation (e.g., *E2f1*, *Ccnd2*, and *Cdkn1a*) (*35*) and prosensory cell fate specification [e.g., *Sox11* (*36*)] (Fig. 4B). Although Atoh1-GFP reporter–positive cells (HCs) are not detected in FST- and LIN28B-overexpressing organoid cultures before 10 to 11 days of expansion, early HC-specific transcripts including transcript for *Atoh1*, *Pou4f3*, and *Gfi1* were significantly up-regulated in FST + LIN28B samples but close to undetectable in FST or LIN28B samples. *Atoh1*, *Pou4f3*, and *Gfi1* encode for HC-specific transcription factors, which are sufficient to reprogram mouse embryonic stem cells or fibroblasts into inner ear HCs (*37*, *38*), suggesting that coactivation of LIN28B and FST may prime progenitor-like cells for HC fate induction. To identify pathways and biological processes that may be altered by coactivation of FST and LIN28B, we performed a gene ontology enrichment analysis using Metascape, a web-based portal (*39*). Genes that function in extracellular structure and extracellular matrix organization were significantly enriched in the list of genes down-regulated in FST + LIN28B samples (fig. S3, C and D, and table S5). By contrast, the list of up-regulated genes was significantly enriched for genes that function in pathways and biological processes associated with self-renewal and growth, with the top pathways being pathways regulating pluripotency of stem cells and cancer (Fig. 4C) and the top biological processes being embryonic morphogenesis and embryonic development (fig. S3, A and B, and table S4).

Reverse transcription quantitative polymerase chain reaction (RT-qPCR) experiments confirmed that the expression of progenitor-specific genes *Hmga2* and *Trim71* was increased in response to LIN28B or FST + LIN28B overexpression (fig. S3E). Similarly, immunostaining and Western blot analysis of HMGA2 protein expression revealed robust induction of HMGA2 in LIN28B and to an even greater extent in FST + LIN28B–overexpressing organoids and little to no induction of HMGA2 protein expression in control and FST-overexpressing organoids (Fig. 4, D, G, and I). Conversely, the SC-specific protein S100A1, which is abundant in Deiter’s cells and pillar cells, was reduced nearly twofold in LIN28B- and FST + LIN28B–overexpressing organoids compared to control but remained unchanged in FST-overexpressing organoids (Fig. 4, E, F, and H). LIN28B-induced down-regulation of S100A1 protein expression and ectopic induction of HMGA2 protein expression in SCs were independently confirmed by permanently labeling Deiters’ cells and pillar cells before culture by making use of *Fgfr3-CreER^T2^* and *ROSA26 ^tdTomato^* transgenes (fig. S4A) (*6*). After 7 days of expansion, tdTomato^+^ Deiters’ cells and pillar cells in control organoid cultures highly expressed S100A1 and lacked HMGA2 expression (fig. S4, B and C). By contrast, tdTomato^+^ Deiters’ cells and pillar cells in LIN28B- or FST + LIN28B–overexpressing organoid cultures lacked S100A1 expression and highly expressed HMGA2 (fig. S4, B and C). Furthermore, we found that tdTomato^+^ Deiters’ cells/pillar cells that overexpressed both FST and LIN28B during expansion produced large clusters of MYO7A^+^Atoh1-nGFP^+^ HCs, while tdTomato^+^ Deiters’ cells/pillar cells that only overexpressed LIN28B formed only few small clusters of HCs (fig. S4D), further validating the importance of FST as an important cofactor for LIN28B-mediated HC production.

### FST counterbalances increase in TGF-β–type signaling induced by LIN28B

**Fig. 5.**
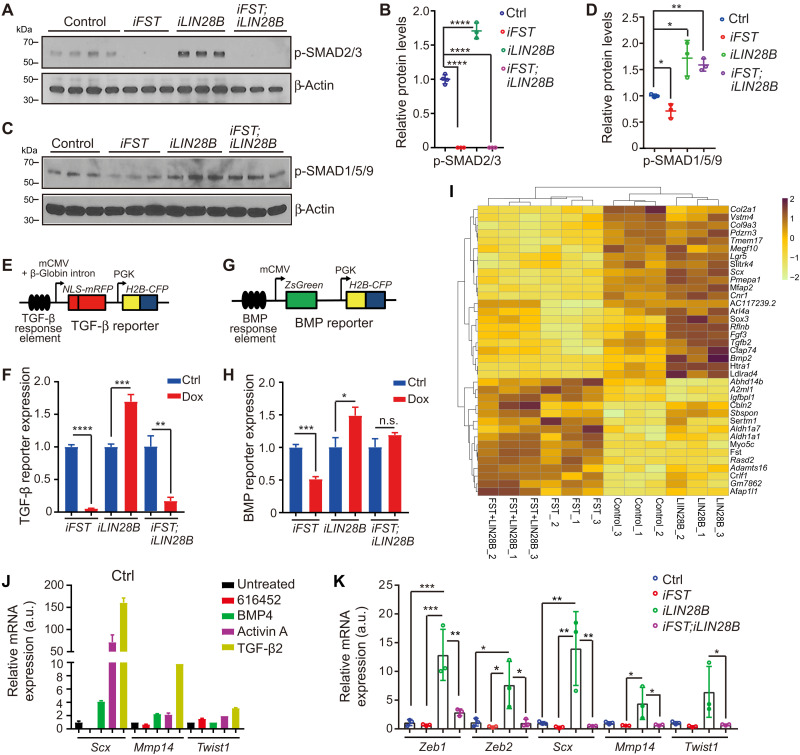
FST counterbalances aberrant activation of TGF-β–type signaling by LIN28B in P5 cochlear organoids. p-SMAD2/3 and p-SMAD1/5/9 (A to D) and TGF-β reporter and BMP reporter (E to H) expression in P5 control and FST-, LIN28B-, and FST + LIN28B–overexpressing organoids after 7 days of expansion (*n* = 3, from one representative experiment and three independent experiments). (**A**) Immunoblots of p-SMAD2/3 and β-actin protein in organoids. (**B**) Normalized p-SMAD2/3 protein in (A). (**C**) Immunoblots of p-SMAD1/5/9 and β-actin protein in organoids. (**D**) Normalized p-SMAD1/5/9 protein in (C). (**E**) TGF-β reporter construct. mCMV, minimal cytomegalovirus promoter; PGK, phosphoglycerokinase promoter; NLS-mRFP, nuclear localized monomeric red fluorescent protein; H2B-CFP, histone 2B-cyan fluorescent protein. (**F**) RT-PCR–based analysis of TGF-β reporter activity. (**G**) BMP reporter construct. ZsGreen, Zoanthus sp. green fluorescent protein. (**H**) RT-PCR–based analysis of BMP reporter activity. (**I**) Heatmap of row *z* scores computed for DEGs between FST and control. (**J**) RT-PCR analysis of mesenchymal marker expression (*Scx*, *Mmp14*, and *Twist1*) in P2 wild-type organoids treated with 616452 (2 μM), BMP4 (50 ng/ml), Activin A (50 ng/ml), and TGF-β2 (50 ng/ml) overnight (*n* = 3). (**K**) RT-PCR analysis of mesenchymal markers (*Zeb1*, *Zeb2*, *Scx*, *Mmp14*, and *Twist1*) in P5 control and FST-, LIN28B-, and FST + LIN28B–overexpressing organoids (*n* = 3). One-way ANOVA with Tukey’s correction was used to calculate *P* values in (B), (D), and (K). *P < 0.05, **P < 0.01, ***P < 0.001, and ****P < 0.0001. Two-tailed, unpaired *t* test was used to calculate *P* values in (F) and (H).

LIN28B-overexpressing organoids showed increased expression of *Tgfbr1*, *Acvr2b*, and *Inhba*, which encode for the type I TGF-β receptor, type II Activin/BMP receptor, and Activin ligand Activin A, suggesting that LIN28B reactivation may augment TGF-β–type signaling (table S2). To address the effect of LIN28B and/or FST overexpression on TGF-β–type signaling, we analyzed the levels of the phosphorylated form of SMAD2, SMAD3 (p-SMAD2/3) and SMAD1, SMAD5 and SMAD9 (p-SMAD1/5/9) in control, FST-, LIN28B-, and FST + LIN28B–overexpressing organoids after seven days of expansion. Activin, TGF-β, and BMP signaling regulate gene expression by receptor-mediated activation (phosphorylation) of regulatory SMAD transcription factors [reviewed in (*40*)]. The presence of p-SMAD2/3 is an indicator for Activin/TGF-β signaling (fig. S5, L and M), while p-SMAD1/5/9 is an indicator for BMP signaling (fig. S5N). Our analysis revealed opposing effects of LIN28B and FST on TGF-β/Activin and BMP signaling. LIN28B increased both TGF-β/Activin and BMP signaling, while FST severely reduced Activin/TGF-β signaling and dampened BMP signaling. We found that p-SMAD1/5/9 and p-SMAD2/3 protein levels were 1.5- to twofold higher in LIN28B-overexpressing organoids compared to control (Fig. 5, A to D). By contrast, p-SMAD2/3 proteins were undetectable in FST- and FST + LIN28B–overexpressing organoids, and p-SMAD1/5/9 protein levels were reduced in FST-overexpressing organoids (Fig. 5, A to D). Stimulation of TGF-β–type signaling in cochlear organoids did not alter Akt-mTOR activity (p-Akt, p-S6, and p-4EBP) (fig. S5, L to N), which is a main target of LIN28B-mediated regulation in stage P5 cochlear organoids and tissue (*16*).

To further investigate the effects of FST and LIN28B on SMAD2/3 and SMAD1/5/9-dependent gene regulation, we infected stage P5 control, *iFST*, *iLIN28B*, and *iFST;iLIN28B* tg cochlear epithelial cells with lentivirus containing a TGF-β or BMP reporter construct (*41*). TGF-β reporter (monomeric red fluorescent protein, mRFP) expression is controlled by a TGF-β response element harboring canonical SMAD2/3 binding sites (Fig. 5E), while BMP reporter expression (Zoanthus sp. green fluorescent protein, ZsGreen) is controlled by a BMP response element harboring canonical SMAD1/5/9 sites (Fig. 5G). We found that TGF-β reporter expression was 1.7-fold up-regulated in LIN28B-overexpressing organoids compared to control, but TGF-β reporter expression was nearly abolished in response to FST overexpression (*iFST* or *iFST;iLIN28B*) (Fig. 5, E and F). Furthermore, the 1.5-fold up-regulation of BMP reporter expression by LIN28B and the twofold down-regulation of BMP reporter expression by FST compared to control were normalized by coactivation of FST and LIN28B (Fig. 5, G and H). How does FST block Activin/TGF-β and BMP signaling? FST is a potent antagonist of Activin signaling. FST directly binds to and sequesters Activin-type ligands, hindering receptor activation (*42*), which, in certain tissues such as the tongue, leads to reduced BMP-type ligand expression (*43*). Among the BMP/TGF-β–type ligands that were significantly reduced in response to FST overexpression were *Bmp2* and *Tgfb2* (Fig. 5I and fig. S5, G and H). Consistent with *Tgfb2* and *Bmp2* being targets of Activin-FST–mediated regulation, we found that Activin A treatment led to robust up-regulation of *Tgfb2* and *Bmp2* expression in P5 cochlear organoids, an effect that was dampened in the presence of FST (fig. S5, I and J). By contrast, the expression of *Bmp4*, a BMP ligand thought to be essential for prosensory cell specification, was unchanged by Activin A treatment and/or FST activation (fig. S5K). In vivo, *Bmp2* is mainly expressed in differentiating outer HCs (*44*), while *Tgfb2* transcript and TGF-β2 protein have been reported to be highly expressed in differentiating and maturing cochlear epithelial cells including SCs (*26*, *45*). Analysis of *Inhba* and *Tgfb2* ligand expression in acutely isolated Lfng-GFP^+^ cochlear SCs stages P1, P5, and P13 revealed that the expression of *Tgfb2* increases by ninefold between P1 and P13, and *Inhba* expression increases by more than 2.5-fold (fig. S5B), revealing a strong correlation between the rising levels of TGF-β–type ligand expression in cochlear SCs and the decline in regenerative capacity. Moreover, our analysis of TGF-β2 protein abundance in cochlear sensory epithelia revealed that the active form of TGF-β2 protein increases between stage P2 and P5 (fig. S5, C and D). Furthermore, the analysis of *Inhba* and *Tgfb2* expression in acutely isolated cochlear sensory epithelia showed that at stages P1 and P5, *Inhba* expression was more than threefold higher in the base than apex, while *Fst* expression was near to undetectable in the cochlear base (fig. S5, E and F), revealing a strong correlation between high Activin signaling in the cochlear base and the inability of basal SCs to generate/regenerate HCs in the early postnatal cochlea.

TGF-β signaling is known to positively regulate the expression of genes that promote epithelial-to-mesenchymal transition (EMT) and cartilage formation including *Scx* (*46*) and *Prrx1* (*47*), which encode for transcription factors and *Mmp14*, which encodes for matrix metalloproteinase involved in extracellular matrix remodeling (*48*) (Fig. 5J). Consistent with FST limiting aberrant induction of TGF-β–type signaling by LIN28B, we found that expression of *Scx* and *Prrx1* was up-regulated in LIN28B-overexpressing samples but significantly down-regulated in FST + LIN28B–overexpressing samples compared to control (Fig. 4B and table S1). After 11 days of expansion, LIN28B-overexpressing organoids expressed *Mmp14*, *Scx*, as well as *Twist1*, *Zeb1*, and *Zeb2*, which encode for EMT-activating transcription factors that act downstream of SCX (*49*), at a 5- to 15-fold higher level than control. By contrast, FST- and FST + LIN28B–overexpressing organoids expressed *Mmp14*, *Scx*, *Twist1*, *Zeb1*, and *Zeb2* maintained normal control levels (Fig. 5K). Collectively, these data suggest that FST may be critical to limit aberrant activation of TGF-β signaling in LIN28B-overexpressing organoids, which, among others, may prevent the conversion of SCs/prosensory cells into mesenchymal cells.

### Endogenous FST is required for LIN28B to stimulate HC formation

**Fig. 6.**
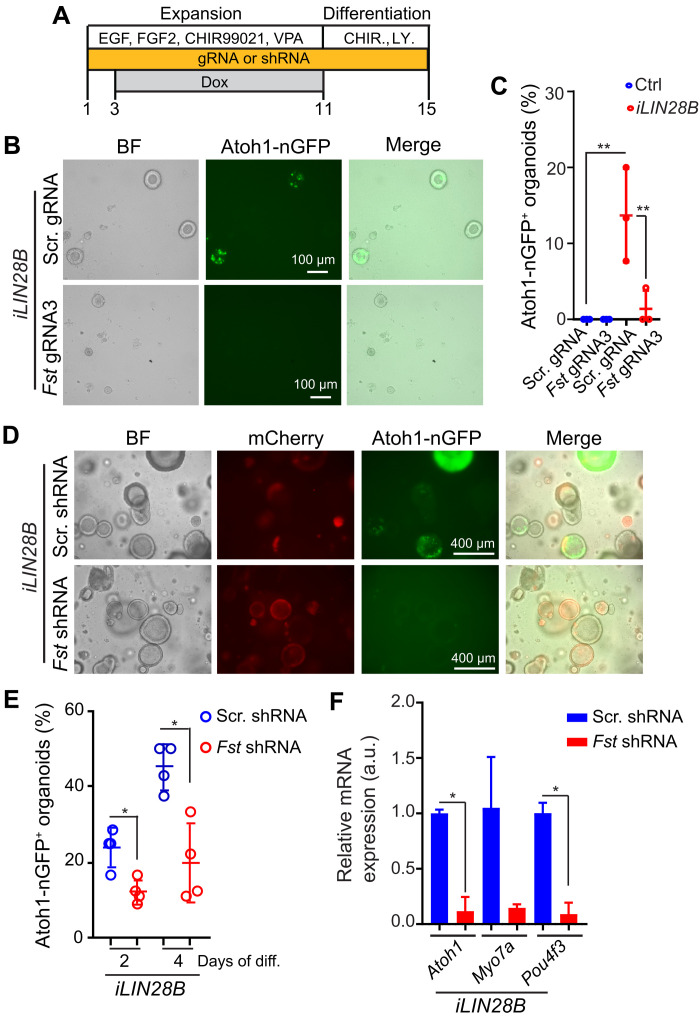
Endogenous *Fst* is required for HC production in LIN28B-overexpressing stage P5 cochlear organoids. (**A**) Experimental scheme. Cochlear organoid cultures were established with cochlear epithelial cells from stage P5 *iLIN28B* tg mice and control littermates. *Atoh1-nGFP* marks nascent HCs. To knockout *Fst*, cochlear epithelial cells were infected with lentivirus expressing Cas9 protein and *Fst*-specific gRNA (Fst-gRNA) (**B** and **C**). To knockdown *Fst*, cochlear epithelial cells were infected with lentivirus expressing mCherry and *Fst*-specific shRNA (**D** to **F**). As control, cochlear epithelial cells were infected with lentivirus expressing nonspecific scramble (scr) gRNA or shRNA. To induce LIN28B overexpression, dox was administered at day 3 of expansion. (B) Bright-field and green fluorescent (Atoh1-nGFP) images of LIN28B-overexpressing organoids transduced with control lentivirus or lentivirus expressing Cas9 and *Fst*-gRNA. (C) Percentage of Atoh1-nGFP^+^organoids in (B) (*n* = 3, two independent experiments). (D) Bright-field, mCherry (red), and green fluorescent (Atoh1-nGFP) images of LIN28B overexpression organoids infected with control or *Fst*-shRNA lentivirus. (E) Percentage of Atoh1-nGFP^+^organoids in (D) after 2 and 4 days of differentiation (*n* = 4, two independent experiments). (F) RT-PCR–based analysis of HC-specific (*Atoh1*, *Myo7a*, and *Pou4f3*) mRNA expression in (D) after 4 days of differentiation (*n* = 3, from one representative experiment and two independent experiments). One-way ANOVA with Tukey’s correction was used to calculate *P* values in (C), and two-tailed, unpaired *t* test was used to calculate *P* values in (E) and (F). *P < 0.05 and **P < 0.01.

To determine whether FST is required for HC production in LIN28B-overexpressing organoids, we used CRISPR-Cas9–based targeting approach to knockout *Fst* in stage P5 cochlear organoid culture (*50*). A Cas9 expression construct and sequence-specific guide RNAs (gRNAs) were delivered using lentiviral particles. A nonspecific scramble (scr) gRNA was used as control. On the basis of pilot experiments conducted using murine P19 cells, we selected *Fst* gRNA3 to knockout *Fst* in cochlear organoid culture (fig. S6, A to C). We infected cochlear epithelia cells from stage P5 LIN28B tg mice and control littermates with *Fst* gRNA3– or *scr* gRNA–expressing lentivirus, and after 3 days of expansion, dox was added to the culture medium to induce LIN28B overexpression (Fig. 6A). We found that *Fst* knockout mildly reduced organoid formation efficiency in control and LIN28B-overexpressing organoid cultures (fig. S6D) and almost completely blocked the formation of Atoh1-nGFP^+^ cell clusters in LIN28B-overexpressing organoid cultures (Fig. 6, B and C). To independently confirm these results, we used short hairpin RNAs (shRNAs) to knockdown endogenous *Fst* expression in stage P5 LIN28B-overexpressing cochlear organoids. Sequence-specific shRNAs or scr shRNA was delivered by lentiviral infection. On the basis of pilot experiments in murine P19 cells, we selected *Fst shRNA3* construct to knockdown *Fst* in cochlear organoid culture (fig. S6, B and C). We infected cochlear epithelia cells from stage P5 LIN28B tg mice with lentivirus that coexpressed mCherry with *Fst* shRNA3 or unspecific scr shRNA and, after 3 days of expansion, added dox to induce LIN28B overexpression (Fig. 6A). As anticipated, *Fst* knockdown in LIN28B-overexpressing organoid culture decreased the number of Atoh1-nGFP^+^ organoids by more than twofold (Fig. 6, D and E). Furthermore, the expression of early HC-specific transcripts (*Aoth1*, *Myo7a*, and *Pou4f3*) was reduced 5- to 10-fold in LIN28B-overexpressing organoids in response to *Fst* knockdown (Fig. 6F). Together, these results indicate that endogenous FST is essential for HC formation in LIN28B-overexpressing organoids.

### TGF-β signaling limits HC formation in response to LIN28B overexpression

**Fig. 7.**
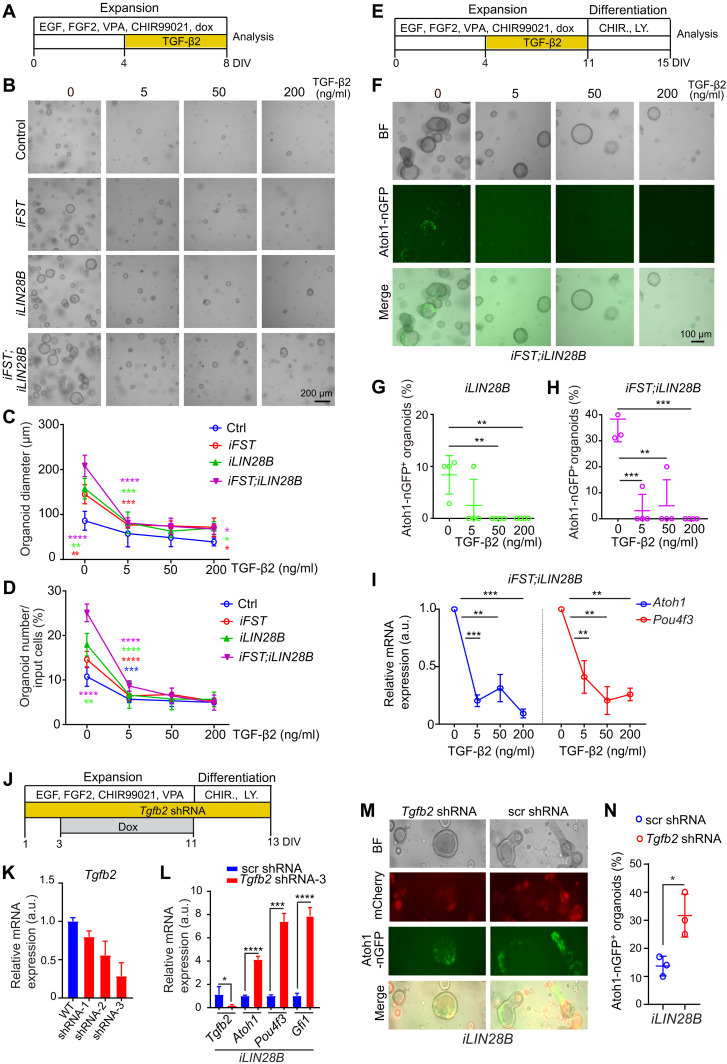
TGF-β2 inhibits HC formation in LIN28B-overexpressing stage P5 cochlear organoids. Organoid cultures were established with cochlear epithelial cells from stage P5 control, *iFST*, *iLIN28B*, and *iFST;iLIN28B* tg mice. (**A**) Experimental scheme (B to D). DIV, day(s) in vitro. (**B**) Low-power bright-field images show that exogenous TGF-β2 inhibits organoid formation and growth. (**C**) Organoid diameter in (B) (*n* = 4, from one representative experiment and three independent experiments). (**D**) Organoid-forming efficiency in (B) (*n* = 4, from one representative experiment and three independent experiments). (**E**) Experimental scheme (F to I). (**F**) Low-power bright-field and green fluorescent (Atoh1-nGFP) images show that exogenous TGF-β2 blocks HC formation in FST + LIN28B–overexpressing cultures. (**G**) Percentage of Atoh1-nGFP^+^ organoids in LIN28B-overexpressing cultures treated with and without TGF-β2 (*n* = 4, from one representative experiment and three independent experiments). (**H**) Percentage of Atoh1-nGFP^+^ organoids in (F) (*n* = 3, untreated group; *n* = 4, TGF-β2–treated groups; from one representative experiment and three independent experiments). (**I**) RT-PCR analyzing *Atoh1* (blue) and *Pou4f3* (red) mRNA induction in (F) (*n* = 3, from one representative experiment and three independent experiments). (**J**) Experimental scheme (K to N). (**K**) RT-qPCR analyzing the *Tgfb2* knockout efficiency using *Tgfb2-*shRNA lentiviral particles. WT, wild-type. (**L**) RT-PCR of HC-specific (*Atoh1*, *Pou4f3*, and *Gfi1*) mRNA expression (*n* = 3, from one representative experiment and two independent experiments). (**M**) Bright-field, mCherry (red), and green fluorescent (Atoh1-nGFP) images of LIN28B-overexpressing organoids infected with control (scr shRNA) or *Tgfb2-*shRNA–expressing lentivirus. (**N**) Percentage of Atoh1-nGFP^+^organoids in (M) (*n* = 3, from one representative experiment and two independent experiments). One-way ANOVA with Tukey’s correction was used to calculate *P* values in (C) to (H). Two-tailed, unpaired *t* test was used to calculate *P* values in (I) to (N). *P < 0.05, **P < 0.01, ***P < 0.001, and ****P < 0.0001.

We next investigated whether overactivation of Activin/TGF-β signaling limits the ability of SCs to generate HCs. We established stage P5 control, FST-, LIN28B-, and FST + LIN28B–overexpressing organoid cultures and added recombinant TGF-β2 (5, 50, and 200 ng/ml) or Activin A (5, 50, and 500 ng/ml) protein to the expansion medium from days 4 to 8 (Fig. 7A and fig. S7A). We found that TGF-β2 (5 ng/ml) reduced organoid formation and organoid growth in FST-, LIN28B, and FST + LIN28B–overexpressing cultures to similar or to below the levels observed in control organoid cultures (Fig. 7, B to D). A qualitative similar inhibitory effect on organoid formation and growth was observed when organoids were exposed to recombinant Activin A protein (50 and 500 ng/ml) (fig. S7, B to D). Furthermore, we found that exposure to recombinant TGF-β2 or Activin A protein during expansion nearly completely blocked HC formation (Atoh1-nGFP^+^ cells) in stage P5 organoids that overexpressed LIN28B or FST + LIN28B (Fig. 7, E to H, and fig. S7, E to H). The inhibitory effect of TGF-β2 or Activin A treatment on HC formation was confirmed by analyzing *Atoh1* and *Pou4f3* transcript expression using RT-PCR (Fig. 7I and fig. S7I). Moreover, we found that exposure to low concentrations of recombinant TGF-β2 protein (5 ng/ml) was sufficient to completely block HC formation in response to Wnt activation and Notch inhibition in stage P5 control and LIN28B-overexpressing cochlear explants (fig. S8).

To determine whether the loss of TGF-β2 function would enhance HC formation in LIN28B-overexpressing organoids, we knocked down *Tgfb2* expression using sequence-specific shRNA constructs (Fig. 7K). As anticipated, knockdown of *Tgfb2* during expansion resulted in a four- to eightfold up-regulation of HC-specific transcripts (*Atoh1*, *Pou4f3*, and *Gfi1*) (Fig. 7, J to L) and a twofold increase in the percentage of HC containing organoids (Atoh1-nGFP) in LIN28B-overexpressing organoids after 2 days of differentiation (Fig. 7, M and N). In summary, our data indicate that maintaining low TGF-β and Activin signaling is essential for allowing mitotic and nonmitotic cochlear HC formation at postnatal stages.

### FST and LIN28B overexpression promotes spontaneous HC regeneration in perinatal mice

**Fig. 8.**
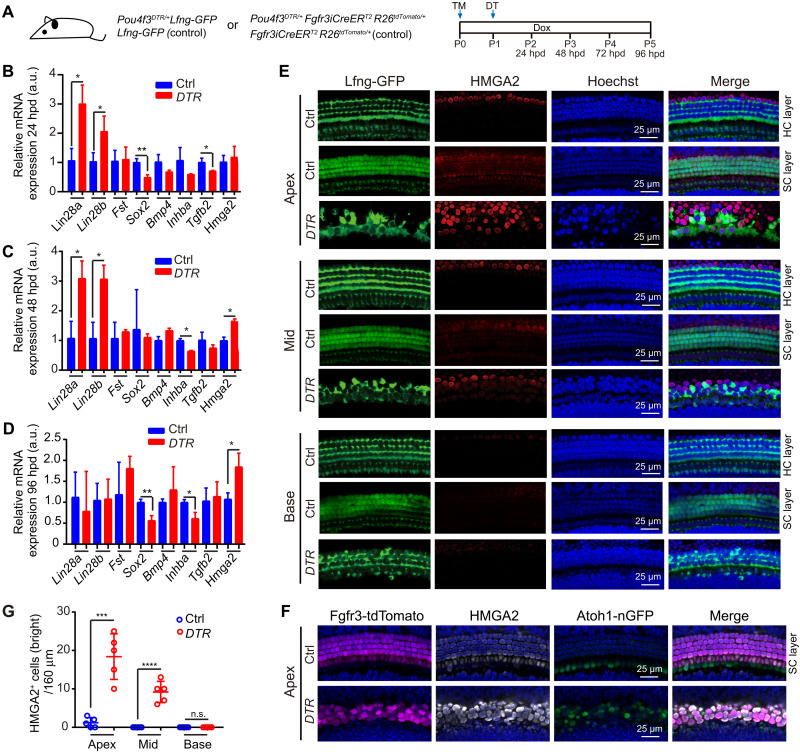
Cochlear HC loss in neonatal mice up-regulates *Lin28a* and *Lin28*b expression. (**A**) Experimental scheme. To ablate HCs, *Pou4f3^DTR/+^* mice and nontransgenic control littermates received DT (6.25 ng/g) at stage P1. To permanently label Deiter’s cells, pillar cells, and outer HCs, *Fgfr3iCreER^T2^;R26*^*tdTomato*/+^ tg mice received 4-hydroxytamoxifen (TM) injection at P0 in (F). (**B** to **D**) RT-PCR–based analysis of the expression of LIN28/*let-7* (*Lin28a*, *Lin28b*, *Hmga2*, and *Sox2*) and TGF-β pathway (*Fst*, *Bmp4*, *Inhba*, and *Tgfb2*) genes in Ctrl (blue) and *Pou4f3^DTR/+^* (DTR, red) cochlear sensory epithelia 24 (B), 48 (C), and 96 (D) hpd injection. (**E**) HMGA2 (red) expression in the HC-damaged (*DTR*) and undamaged Ctrl cochlear sensory epithelia in the apex, mid, and base 72 hours after DT injection. SC and HC layers are shown for undamaged control tissue. Lfng-GFP reporter expression marks SCs (green). (**F**) High-power images of tdTomato (magenta), HMGA2 (gray) and Atoh1-nGFP (green) expression in control and *Pou4f3^DTR/+^* tg sensory epithelia 72 hpd injection. (**G**) Quantification of bright HMGA2^+^ cells within the cochlear sensory domain in (E) and (F). Two-tailed, unpaired *t* test was used to calculate *P* values in (B) to (D) and (G). *P < 0.05, **P < 0.01, ***P < 0.001, and ****P < 0.0001.

Recent studies revealed that severe HC loss in neonatal mice stimulates some few SCs located in the cochlear apex to reenter the cell cycle and form new HCs (*3*). To determine whether such “spontaneous” HC regeneration may be linked to an increase in *Lin28b* expression and/or a reduction in TGF-β–type signaling, we ablated HCs in neonatal mice that were expressing the human diphtheria toxin receptor (DTR) under the control of HC-specific gene *Pou4f3* (*Pou4f3^DTR/+^*) transgene, which renders murine HCs sensitive to diphtheria toxin (DT) (*51*). In our first set of experiments, we administered DT to stage P1 *Pou4f3^DTR/+^* tg mice and control littermates, enzymatically isolated their cochlear sensory epithelia 24, 48, and 96 hours post-DT (hpd) injection, and used RT-qPCR to analyze the expression of genes associated with the LIN28/*let-7* pathway (*Lin28a*, *Lin28b*, *Hmga2*, and *Sox2*) and TGF-β–type signaling (*Fst*, *Inhba*, and *Tgfb2*) (Fig. 8A). We found that at 24 hpd, *Lin28b* and *Lin28a* expression was up-regulated by two- and threefold, while the expression of *Tgfb2* and *Sox2*, which we previously found to be negatively regulated by LIN28B, was down-regulated by 1.5- and 2-fold in HC-damaged (DTR) epithelia compared to undamaged control (Ctrl) epithelia (Fig. 8B). One day later, at 48 hpd, *Lin28b* and *Lin28a* expression remained elevated, and the expression of their downstream effector *Hmga2* was increased by 1.5-fold in the HC-damaged epithelia, while *Inhba* expression was reduced by twofold compared to control (Fig. 8C). At 96 hpd, *Lin28a* and *Lin28b* expression levels were back to control levels, while *Hmga2* expression continued to be elevated and *Inhba* expression continued to be reduced in HC-damaged epithelia compared to control (Fig. 8D). To determine which cells within the HC-damaged cochlear sensory epithelium were up-regulating *Hmga2* expression, we administered DT to stage P1 *Lfng-GFP*; *Pou4f3^DTR/+^* tg mice and *Lfng-GFP* tg control littermates. At 72 hpd, we collected HC-damaged (DTR) and undamaged cochlear sensory epithelia (Ctrl) and stained for HMGA2 protein. Our analysis revealed that HMGA2 induction within the cochlear sensory epithelium follows an apex-to-base gradient, with HMGA2^+^ cells being most frequent in the apex and about half as frequent in the mid turn and absent from the base (Fig. 8, E and G). Furthermore, we found that cells that expressed HMGA2 at a high level lacked Lfng-GFP expression, suggesting that these HMGA2^+^Lfng-GFP^−^ cells may represent SCs that underwent some form of dedifferentiation (Fig. 8E). In the early postnatal cochlea, throughout the apex, HMGA2 continues to be expressed in a band of cells adjacent to outer HCs. To rule out that the observed HMGA2^+^ cells originated outside the sensory epithelium, we permanently labeled Deiter’s cells, pillar cells, and outer HCs before DT-induced HC ablation in stage P1 *Atoh1-nGFP*;*Fgfr3iCreER^T^;R26^Tdtomato/+^;Pou4f3^DTR/+^* tg mice. The *Atoh1-nGFP* reporter allowed us to distinguish between outer HCs (Atoh1-nGFP^+^tdTomato^+^) and Deiter’s cells/pillar cells (Atoh1-nGFP^–^ tdTomato^+^). Our lineage tracing experiment confirmed that the majority of bright HMGA2-expressing cells in HC-damaged cochlea are of Deiter’s cells/pillar cell origin (Fig. 8F). Moreover, HC ablation experiments in control, FST-, and/or LIN28B-overexpressing mice showed that the number of SOX2^+^ cells that highly expressed HMGA2 in response to HC damage was further increased by overexpression of LIN28B and FST (fig. S9, A to C). However, in the absence of HC damage, LIN28B and FST overexpression failed to induce HMGA2 protein expression in SCs, indicating that in vivo, without damage, tg *LIN28B* and *FST* expression may be too low to counter high *let-7* expression and high TGF-β signaling within the cochlear sensory epithelium.

**Fig. 9.**
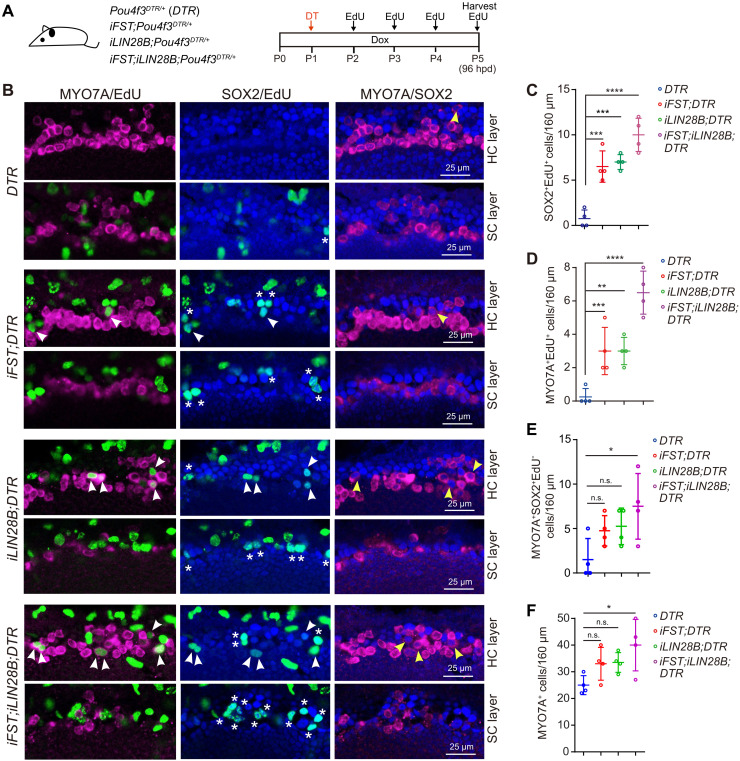
FST and LIN28B overexpression enhances cochlear SC proliferation and mitotic HC regeneration in neonatal mice. (**A**) Experimental scheme. To induce *FST* and/or *LIN28B* transgene expression in neonatal mice, nursing dams received dox-containing feed starting at P0 until tissue harvest at P5. To ablate HCs, mice received DT (6.25 ng/g) at stage P1. To label dividing cells, mice received single injections of EdU (25 ng/ml) at P2, P3, P4, and P5. (**B**) High-power images of HC-damaged cochlear sensory epithelia isolated from control, FST (*iFST*)–, LIN28B (*iLIN28B*)–, and FST + LIN28B (*iFST;iLIN28B*)–overexpressing mice stage P5 stained for MYO7A (magenta), EdU (green), and SOX2 (blue). HC and SC layers at the cochlear mid-apex are shown. Asterisks indicate EdU^+^SOX2^+^ cells, white arrowheads indicate EdU^+^MYO7A^+^ cells, and yellow arrowheads indicate MYO7A^+^SOX2^+^EdU^–^ cells. (**C**) Quantification of SCs that reenter the cell cycle (SOX2^+^EdU^+^) in (B). (**D**) Quantification of HCs that were regenerated through a mitotic mechanism (MYO7A^+^EdU^+^) in (B). (**E**) Quantification of HCs that were regenerated through a nonmitotic mechanism (MYO7A^+^SOX2^+^EdU^–^) in (B). (**F**) Quantification of total number of HCs (MYO7A^+^) in (B). Please note that SC proliferation and HC regeneration were limited to the apical and mid-apical region. One-way ANOVA with Tukey’s correction was used to calculate *P* values. *P < 0.05, **P < 0.01, ***P < 0.001, and ****P < 0.0001.

We next investigated whether FST and/or LIN28B overexpression would enhance spontaneous cochlear HC regeneration in neonatal mice. We administered DT to stage P1 *Pou4f3^DTR/+^* tg mice that also carried the *iFST* and/or *iLIN28B* transgene and their control littermates. To be able to capture dividing SCs, we administrated EdU daily until tissue harvest at P5 (Fig. 9A). Our analysis, which was limited to the cochlear apical region, revealed that overexpression of FST (*iFST;DTR*) and overexpression of LIN28B (*iLIN28B;DTR*) enhanced the frequency by which SCs reentered the cell cycle (SOX2^+^EdU^+^) (Fig. 9, B and C) and enhanced the frequency of mitotic HC regeneration (MYO7A^+^EdU^+^) (Fig. 9, B and D). However, FST overexpression by itself or LIN28B overexpression by itself failed to boost the rate of nonmitotic HC regeneration and failed to significantly increase the total number of HCs (Fig. 9, B, E, and F). By contrast, we found that coactivation of FST + LIN28B (*iFST;iLIN28B;DTR*) significantly increased both the rate of nonmitotic HC regeneration and the total number of HCs compared to control tissue (DTR). Moreover, we found that FST + LIN28B–overexpressing cochlear tissue showed the highest rate of SC proliferation and mitotic HC regeneration of all conditions examined (Fig. 9, B to F).

**Fig. 10.**
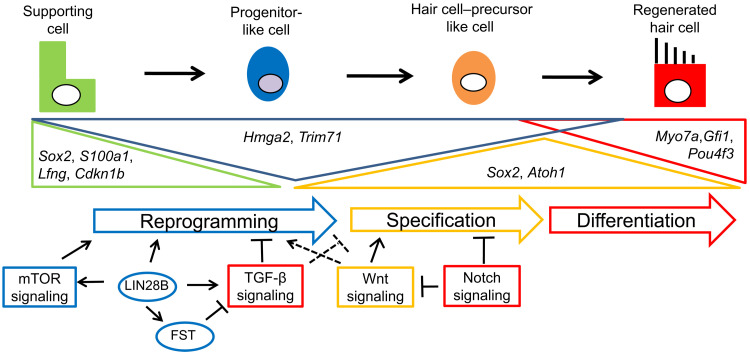
Working model for how LIN28B and FST regulate SC reprogramming and HC regeneration. We propose that HC regeneration occurs in three steps: (i) SC reprogramming, (ii) HC fate specification, and (iii) HC differentiation. In the presence of regenerative cues (e.g., HC loss and high Wnt signaling), activation of LIN28B and suppression of TGF-type signaling by FST trigger the dedifferentiation of SCs into progenitor-like cells and primes them for HC fate induction. The reprogramming phase is characterized by partial down-regulation of SC-specific genes (*Sox2*, *S100a1*, *Lfng*, and *Cdkn1b*) and reinduction of select progenitor-specific genes (*Hmga2* and *Trim71*). High Wnt activity and low Notch signaling are required to induce early HC-specific genes in “progenitor-like cells” during the specification phase. The final, differentiation phase is characterized by gradual down-regulation of progenitor-specific genes and up-regulation of HC-specific genes.

We lastly examined whether low TGF-β signaling is a requirement for spontaneous HC regeneration. To address this question, we damaged cochlear HCs in stage P1 control and LIN28B + FST (*iFST;iLIN28B*)–overexpressing cochlear explants using gentamicin and cultured the tissue for 4 days with or without exogenous TGF-β2 protein (fig. S10A). We found that without TGF-β2, FST + LIN28B overexpression significantly enhanced the rate of spontaneous HC regeneration and increased the total number of HCs throughout the length of the cochlear explant (fig. S10, B to D). By contrast, we found that in the presence of TGF-β2, spontaneous HC regeneration was completely abolished in control explants and significantly reduced in FST + LIN28B–overexpressing explants (fig. S10, B to D). On the basis of our findings, we propose that suppression of TGF-β–type signaling is essential for LIN28B-mediated reprogramming of cochlear SCs into progenitor-like cells, as well as subsequent HC fate induction and HC formation (Fig. 10).

## DISCUSSION

Millions of people worldwide suffer from hearing deficits and deafness caused by the loss of cochlear HCs. In mammals, cochlear HCs are not regenerated, and their loss due to loud noise, disease, or aging is permanent. Recent studies conducted in mice indicate that cochlear SCs have some latent capacity to form HCs, making SCs a promising target for cell-based strategies to combat hearing loss. However, while immature cochlear SCs readily respond to HC fate–inducing cues (severe HC loss, ectopic *Atoh1* expression, inhibition of Notch signaling, and activation of Wnt/β-catenin signaling), attempts to stimulate functional mature cochlear SCs to generate/regenerate HCs under similar conditions yielded thus far no or only very limited success (*9*, *10*, *12*, *14*, *15*, *21*). Our data show that as the cochlear sensory epithelium starts to undergo maturation (stage P5 in mice), SCs fail to reenter the cell cycle and form HCs even under the most optimized and ideal conditions such as an organoid culture platform. We further show that such resistance to mitotic and HC fate–inducing cues can be reversed by the transient reactivation of the RNA binding protein LIN28B and the Activin antagonist FST, allowing for the production of new HCs by mature SCs.

Regenerative processes involving terminal differentiated cells frequently involve a dedifferentiation step, in which cells revert to a more primitive progenitor-like state (*52*). Similar to their mammalian counterparts, SCs in birds are highly specialized postmitotic cells. The lack of progenitor-like features has led to the yet to be proven hypothesis that a partial dedifferentiation of SCs, in which SCs revert to a transitional progenitor-like state, may be an essential part of the HC regenerative process [reviewed in (*53*)]. Our data provide evidence that mammalian cochlear SCs that begin to undergo maturation (stage P5 in mice) have the capacity to partially dedifferentiate and activate a progenitor-like state and that such reprogramming step is an essential part of the HC regenerative process. Our global transcriptome data reveal that coactivation of LIN28B and FST in cultured cochlear epithelial cells (organoids) triggers the down-regulation of SC-specific genes involved in connective tissue and extracellular matrix formation and leads to the up-regulation of genes essential for embryonic/inner ear morphogenesis and HC fate induction. We validated the FST- and LIN28B-induced SC reprogramming by analyzing SC marker (S100A1, Lfng-GFP) and progenitor marker (HMGA2) expression in FACS-isolated and/or lineage-traced SCs in vitro and in vivo.

How do LIN28B and FST reprogram SCs into HC progenitor-like cells? Our data indicate that LIN28B and FST contribute to SC reprogramming through distinctly different mechanisms. LIN28B is a potent repressor of *let-7* miRNAs, which are a main barrier to self-renewal and cell reprogramming (*54*). Reflecting such function, we found that LIN28B reactivation increased the expression of *let-7* target genes critical for stemness (*Arid3a*, *Hmga2*, and *Igf2bp1*) (*25*, *55*, *56*), primed pluripotency (*Trim71* and *Hmga2*) (*57*, *58*), and stem cell metabolism such as increased glycolysis (*Hk2* and *Igf2bp2*) (*59*, *60*). Such metabolic reprogramming is consistent with our recent finding that LIN28B boosts the regenerative competence of cochlear SCs through the activation of phosphatidylinositol 3-kinase (PI3K)–mTOR signaling (*16*). In addition, we found that LIN28B reactivation led to the down-regulation of SC-specific transcription factors including members of the nuclear factor I (NFI) transcription factor family (*Nfic* and *Nfix*). In the retina, NFIA, NFIB, and NFIX promote Müller glia cell specification and differentiation (*32*), and their loss enables Müller glia cells to proliferate and produce bipolar- and amacrine-like cells in adult mice after injury (*61*). Future studies are warranted to determine whether NFIC and NFIX play a similar role in cochlear SC development and cochlear SC plasticity.

In various cell types including epithelial and glial cells, activation of TGF-β signaling induces the expression of cyclin-dependent kinase inhibitors (CDKIs). High expression of CDKIs such as p21Cip1 (CDKN1A) and p15Ink4b (CDKN2B) triggers proliferative quiescence and senescence (*62*, *63*), which are considerable barriers to cell/tissue regeneration. In addition, TGF-β–induced EMT can lead to excessive deposition of extracellular matrix proteins and tissue fibrosis, which has been shown to interfere with tissue repair and regeneration in various organ systems including the kidney, lung, and liver (*64*). On the basis of our data, we propose that coactivation of FST enhances LIN28B-induced SC reprogramming through limiting aberrant activation of TGF-β signaling. In support of such model, we find that FST coactivation reverses LIN28B’s induced increase in TGF-β/Activin activity (p-SMAD2/3 activity). Furthermore, we show that FST coactivation prevents the ectopic induction of EMT promoting transcription factors ZEB1 and ZEB2 and limits the up-regulation of p21Cip1 and p15Ink4b that is observed in LIN28B alone. How does FST interfere with TGF-β signaling? Unlike Activin-type ligands, TGF-β ligands are not direct targets of FST repression. Instead, we find that FST inhibits the transcriptional activation of *Tgfb2* by Activin A and show that exogenous TGF-β2 or knockdown of endogenous *Fst* block HC production in LIN28B-overexpressing organoids, while knockdown of endogenous *Tgfb2* or FST overexpression enhances LIN28B-induced HC production.

Damage to HCs triggers some limited spontaneous HC regeneration within the cochlear apex in neonatal mice (*3*). The cellular and molecular mechanisms that facilitate such regenerative plasticity are largely unknown. The provided data indicate that LIN28A/B reactivation and TGF-β signaling suppression are part of an endogenous repair mechanism that facilitates spontaneous HC regeneration. We show that HC damage triggers the up-regulation of *Lin28a/b* and the down-regulation of *Tgfb2* mRNAs within the cochlear sensory epithelium, which is followed by the up-regulation of progenitor-specific HMGA2 expression and the down-regulation of SC-specific Lfng-GFP expression. Furthermore, we demonstrate that the low level of SC proliferation and HC regeneration that is normally observed following HC damage is greatly enhanced by transgenic coactivation of LIN28B and FST but abolished by activation of TGF-β signaling.

Mounting evidence suggests that induction of *Lin28a* and/or *Lin28b* expression in response to injury is part of an evolutionary conserved repair mechanism. For example, retinal injury in zebrafish triggers the up-regulation of *Lin28a* in Müller glia cells, which is essential for the reprogramming of Müller glia cells into proliferative progenitor-like cells that are capable of regenerating all major retinal cell types (*65*). Similarly, a recent study found that severe damage in the zebrafish lateral line leads to induction of *lin28a* expression by the transcription factor yap1 and that *lin28a* function is essential for the reestablishment of the HC progenitor pool and subsequent HC regeneration through promoting Wnt signaling (*66*). Ectopic YAP activation in conjunction with severe HC damage triggers cochlear SC proliferation in stage P10 mice (*67*). It will be of interest to determine whether YAP activation in mice induces *Lin28a* and/or *Lin28b* expression in cochlear or vestibular SCs. Low Notch signaling and/or high Wnt signaling is a requirement for spontaneous cochlear HC regeneration in neonatal mice (*68*, *69*). Future work will address how LIN28A/B and TGF-β signaling intersect with Notch and Wnt signaling pathway during HC regeneration.

It needs to be noted that without severe HC damage, LIN28B and/or FST overexpression is not sufficient to enhance SC reprogramming and stimulate subsequent HC regeneration in neonatal mice or neonatal cochlear explants. Our cochlear organoid and explant experiments indicate that activation of FGF2/EGF and Wnt signaling is a prerequisite for LIN28B-FST–mediated SC reprogramming. It will be of interest to further explore whether and to what extent HC death may activate these pro-growth signaling pathways in neonatal cochlear SCs. Moreover, it needs to be determined whether overexpression of LIN28B and FST in the HC-damaged cochlea of mice stage P5 or older would facilitate SC dedifferentiation and whether LIN28B and FST expression when combined with HC fate–inducing factors (e.g., ATOH1 activation) would enable HC regeneration.

## MATERIALS AND METHODS

### Mouse breeding and genotyping

All experiments and procedures were approved by the Johns Hopkins University Institutional Animal Care and Use Committees protocol, and all experiments and procedures adhered to National Institutes of Health–approved standards. The *Atoh1-nGFP* tg (tg) mice were obtained from J. Johnson (University of Texas Southwestern Medical Center, Dallas) (*23*). *Fgfr3-iCreER^T2^* tg mice (*70*) were obtained from W. Richardson (University College, London). *Lfng-GFP* tg mice were obtained from N. Heintz (Rockefeller University, New York) (*71*). The *Col1a1-TRE-LIN28B* (*72*) tg mice were obtained from G. Q. Daley (Children’s Hospital, Boston). The *TRE-FST-288* tg mice were obtained from S.-J. Lee (the Jackson Laboratory, Farmington) (*73*). R26^rtTA*^^M2^(no. 006965), *R26^tdTomato/+^* (Ai14) (no. 007914), and *Pou4f3^DTR/+^* (no. 028673) mice were purchased from the Jackson Laboratories (Bar Harbor, ME). We crossed *TRE-FST-288 tg;Col1a1-TRE-LIN28B tg* males/females with ROSA26^rtTA*^^M2/rtTA^^*M2^males/females to obtain *iLIN28B* (Col1a1-TRE-LIN28B tg;R26^rtTA*^^M2/+^), *iFST* (TRE-FST-288 tg;R26^rtTA*^^M2/+^), *iFST;iLIN28B* (TRE-FST-288 tg; Col1a1-TRE-LIN28Btg; R26^rtTA*^^M2/+^), and control littermates (R26^rtTA*^^M2/+^). Mice were genotyped by PCR as previously published. Genotyping primers are listed in table S6. Mice of both sexes were used in this study. Embryonic development was considered as E0.5 (embryonic day 0.5) on the day a mating plug was observed. All animal work was performed in accordance with the approved animal protocols from the Institutional Animal Care and Use Committees at the Johns Hopkins University School of Medicine.

### In vivo FST and/or LIN28B transgene activation and HC ablation

To induce *FST* and/or *LIN28B* transgene expression in *iLIN28B* and/or *iFST* mice, dox was delivered to time-mated females via ad libitum access to feed containing dox (2 g/kg; Bio-Serv, no. F3893). To be able to ablate HCs, *Pou4f3^DTR/+^* mice and littermate control mice received a single dose of DT (6.25 ng/g; Sigma-Aldrich, no. D0564) by intraperitoneal injection at P1.

### Lineage tracing of SCs

To label Deiter’s cells and pillar cells in *Fgfr3iCreER* and *R26^tdTomato^*, tg mice received 4-hydroxytamoxifen (TM) (0.125 mg/g body weight; Sigma-Aldrich, no. H7904) by intraperitoneal injection. For lineage tracing experiments in organoid culture, TM was injected at P4/P5, and tissue was harvested at P6. For lineage tracing experiments in vivo, TM was injected at P0, 24 hours before DT injection at P1.

### Lentiviral vectors and lentivirus production

To delete the mouse *Fst* gene, annealed oligonucleotides for *Fst*-specific CRISPR gRNA sequences *Fst*-gRNA1, *Fst*-gRNA2, and *Fst*-gRNA3 were cloned into the vector LentiCRISPRv2 (Addgene, plasmid #52961) by digesting at Bsm BI restriction site. The sequences of the three gRNAs are as follows: *Fst-*gRNA1, 5′-AGC GGC CGT TCT TTG CTT GG-3′; *Fst-*gRNA2, 5′-TGG CTC CGC CAA GCA AAG AA-3′; *Fst-*gRNA3, 5′-TCT TAT ACA GGA CCT GGC AG-3′. For the gRNA lentivirus packaging, 293T cells are transfected with 12 μg of modified LentiCRISPRv2, 9 μg of psPAX2 (Addgene, catalog no. 12260), and 3 μg of pMD2.G (Addgene, catalog no.12259) plasmids, respectively. After 48 hours, the supernatant was harvested and centrifuged with 25,000 revolutions per minute (RPM) at 4°C for 2.5 hours, and the virus pellets was resuspended in Dulbecco’s Modified Eagle Medium: Nutrient Mixture F-12 (DMEM)/F12 to get the high titer virus. The lentiviral vectors for TGF-β (plasmid) and BMP (plasmid) reporters were provided by E. Fuchs at the Rockefeller University (*41*). For *Fst* and *Tgfb2* knockdown experiments, shRNA sequence was inserted into pLV[shRNA]-mCherry:T2A (VectorBuilder, vector ID: VB201006-1173mjc). The sequence of shRNAs are as follows: scr-shRNA-mCherry, 5′-CCT AAG GTT AAG TCG CCC TCG-3′; *Fst*-shRNA1-mCherry, 5′-CCC AAC TGC ATC CCT TGT AAA-3′; Fst-shRNA2-mCherry, 5′-GGC ATG GAG AGA TGG TCAT TT-3′; *Fst-*shRNA3-mCherry, 5′-TGT CGA ATG AAC AAG AAG AAT-3′; *Tgfb2*-shRNA1-mCherry, 5′-AGC CTG TAC AAC ACC ATA AAT-3′; *Tgfb2*-shRNA2-mCherry, 5′-TACT ACG CCA AGG AGG TTT AT-3′; *Tgfb2*-shRNA3-mCherry, 5′- CCC TCG ACA TGG ATC AGT TTA-3′. For packaging TGF-β/BMP reporters, *Fst*-shRNAs and *Tgfb2*-shRNA, 293T cells are transfected with 20 μg of transfer plasmid, 2 μg of pMDLg/pRRE (Addgene, catalog no. 12251), 2 μg of pRSV-Rev (Addgene, catalog no. 12253), and 2 μg of pCMV-VSV-G (Addgene, catalog no. 8454), respectively. After 48 hours, the supernatant was harvested and centrifuged with 25,000 RFP at 4°C for 2.5 hours, and the virus pellets was resuspended in DMEM/F12 to get the high titer virus.

### Explant culture

Cochlear sensory epithelia including innervating neurons (cochlear explants) from stage P0 or stage P5 mice were isolated by microdissection, and epithelia were cultured on SPI-Pore membrane filters (Structure Probe, no. E1013-MB) in DMEM/F12 (Corning, no. 10–092-CV) containing 1× N-2 (Thermo Fisher Scientific, no.17502048), EGF (5 ng/ml; Sigma-Aldrich, no. SRP3196), and penicillin (100 U/ml; Sigma-Aldrich, no. P3032) as previously described (*16*). To induce *FST* and/or *LIN28B* transgene expression, pregnant/nursing dams received feed containing dox (2 g/kg) starting at E18.5 until tissue harvest. To maintain *FST* and/or *LIN28B* transgene expression, culture media were supplemented with dox hyclate (10 μg/ml; Sigma-Aldrich, no. D9891). To ablate HCs, gentamicin sulfate (100 μg/ml; Sigma-Aldrich, no. G1272) was added to cochlear tissue at plating as previously described (*16*). To induce HC formation in undamaged cochlear explants, culture medium contained CHIR99021 (3 μM; Sigma-Aldrich, no. SML1046) and LY411575 (5 μM; Sigma-Aldrich, no. SML0506). In a subset of experiments, mouse recombinant TGF-β2 (5 ng/ml; R&D Systems, 7346-B2-005) was added to the culture medium.

### Fluorescence-activated cell sorting

For stage P1 and P5 mice, cochlear epithelia were collected separately for each animal, enzymatically purified, and reduced to single cells as previously described (*16*). For stage P13 mice, cochleae for each animal were collected separately, incubated in TrypLE solution (Thermo Fisher Scientific, no. 2604013), triturated, and filtered through a 35-μm filter. Resulting single cells were resuspended in expansion medium, incubated with propidium iodide (PI), and sorted on a MoFlo Legacy sorter with 100-μm nozzle tip. Lfng-GFP^+^, PI^−^cells were collected in expansion medium and cultured as described below.

### Organoid culture

Organoid cultures were established with enzymatically purified cochlear epithelia cells or FACS-isolated SCs. Cells were plated in a drop of Matrigel matrix at high density (2000 cells, stage P5; 3000 cells, stage P13) as previously described (16). To promote organoid formation and growth, the culture medium [DMEM/F12, 1× B27 (Thermo Fisher Scientific, no.12587010), 1× N-2, and penicillin (100 U/ml)] was supplemented with EGF (50 ng/ml), FGF2 (50 ng/ml; Thermo Fisher Scientific, no. PHG0264), CHIR99021 (3 μM), and VPA (1 mM; Sigma-Aldrich, no. P4543). To induce differentiation, the culture medium contained CHIR99021 (3 μM) and LY411575 (5 μM). To induce transgene expression, dox hyclate (0.5 μg/ml) was added to expansion medium. To manipulate Activin, TGF-β, or BMP signaling, recombinant human Activin A (R&D Systems, 338-AC-010/CF), recombinant mouse TGF-β2 (R&D Systems, 7346-B2-005), recombinant human BMP4 (R&D Systems, 314-BP-010), or TGFBR1 inhibitor 616452 (Millipore, 446859-33-2) were used at indicated concentrations. For p-SMAD2/3 and p-SMAD1/5/9 assays, TGF-β–type agonists/antagonists were added to the culture medium overnight, and the following day, organoids were harvested and processed for immunoblotting. For HC formation assays, recombinant TGF-β2 was added to the culture medium for 4 days during expansion. For TGF-β and BMP reporter assay, *iFST*, *iLIN28B*, and *iFST*;*iLIN28B* stage P5 tg cochlear epithelial cells were infected with lentivirus containing TGF-b or BMP reporters before plating, after which infected cells were divided equally into two wells for organoid culture. After 3 days of expansion, dox was added to one well to induce FST and/or LIN28B, the second, no dox well functioned as control. Four days later, relative mRFP (mRFP/H2B-CFP) (TGF-β reporter) and relative ZsGreen (ZsGreen/H2B-CFP) (BMP reporter) transcript levels were analyzed using RT-PCR (see below).

### RNA extraction and RT-qPCR

Organoids were harvested using the Cell Recovery Solution. Total RNA from organoids/tissue was extracted using the miRNeasy Micro Kit (QIAGEN, no. 217084). mRNA was reverse-transcribed into cDNA using the iScript cDNA synthesis kit (Bio-Rad, no. 1708889). qPCRs were carried out in triplicate on a CFX-Connect Real-Time PCR Detection System using SYBR Green Master Mix reagent (Thermo Fisher Scientific, no. 4385612). Gene-specific primers used are listed in table S7. *Rpl19* was used as an endogenous reference gene. Relative gene expression was calculated using ΔΔ*C*_t_ method.

### RNA sequencing and data analysis

For each condition, three independent samples from three different animals were analyzed. Cochlear epithelial cells from stage P5 control, *iFST*, *iLIN28B*, and *iFST*;*iLIN28B* tg mice were used as starting material. Total RNA from control, FST-, LIN28B-, and FST + LIN28B–overexpressing organoids was isolated after 7 days of culture as described above. Samples were processed using an Illumina’s TruSeq Stranded Total RNA kit, per the manufacturer’s recommendations, using unique dual indexes (UDI). The samples were sequenced on the NovaSeq 6000, paired end, 2 × 50–base pair reads to an average depth/sample of 30 million reads. Kallisto (v0.46.1) (*28*) was used to pseudo-align reads to the reference mouse transcriptome and to quantify transcript abundance. The transcriptome index was built using the Ensembl *Mus musculus* v96 transcriptome. The companion analysis tool sleuth was used to identify DEGs (*29*). Sleuth estimates technical gene variance among individual samples using transcript abundance and results from kallisto bootstrapping analysis. We used sleuth to fit two models to the data, a full model in which the condition (overexpression of FST, LIN28B, or both) is accounted for, and a reduced model in which condition is not used as an explanatory variable for variance. We then performed a likelihood ratio test to identify genes for which the condition can account for variance. Last, we performed multiple Wald tests, comparing each condition to the control condition, which outputs a directional effect size for each individual gene. The natural log of effect size, termed the β value, is related but not equivalent to fold change, as it connotes the same ranking and directionality. Wald tests were used to produce a list represented graphically using sleuth along with pheatmap and ggplot2 packages in R (version 4.0.3). Gene identifier conversion, gene annotation, and enrichment analysis were conducted using Metascape (*39*).

### Quantification of organoid formation efficiency, organoid diameter, and percentage of GFP^+^ organoids

Low-power bright-field and fluorescent images of organoid cultures were captured with an Axiovert 200 microscope using 5× and 10× objectives (Carl Zeiss Microscopy). The organoid formation efficiency and the diameter of organoids were measured as previously described (*16*), and the average value per animal was reported. To establish the percentage of GFP^+^ organoids per culture, the total number of organoids and the number of GFP^+^ organoids were established by counting manually. For each genotype and treatment, three independent organoid cultures from three different animals were established and analyzed. At a minimum, two independent experiments were conducted and analyzed.

### Immunohistochemistry

Organoids/cochlear explants were fixed in 4% paraformaldehyde for 30 min, permeabilized and blocked with 0.25% Triton X-100/10% fetal bovine serum, and immunostained as previously described (*16*). Used antibodies are listed in table S8.

### Cell proliferation

For in vitro experiments, EdU (Thermo Fisher Scientific, no. C10338) was added to culture medium at a final concentration of 3 μM. For in vivo experiments, EdU was injected daily intraperitoneal at 25 mg/kg starting at stage P2 until tissue harvest. After cell/tissue harvest and processing for immunohistochemistry, EdU incorporation was detected using the Click-iTPlus EdU Cell Proliferation Kit (Thermo Fisher Scientific, no. C10638) following the manufacturer’s recommendations.

### Cell counts

High-power confocal single-plane and *z*-stack images of fluorescently immunolabeled organoids and explants were taken with 40× objective using LSM 700 confocal microscope (Zeiss Microscopy). To quantify cells in organoid cultures/cochlear explants, three independent fields were randomly selected and analyzed and the average value per animal was reported. A minimum of two independent experiments were conducted in which, at a minimum, three organoid cultures/cochlear explants per genotype and treatment were analyzed and reported.

### Immunoblotting

Organoids were lyzed with radioimmunoprecipitation assay buffer supplemented with protease inhibitor and phosphatase inhibitors and immunoblots conducted as previously described (*16*, *20*). The resulting chemiluminescence was captured using x-ray films. The antibodies used are listed in table S9. ImageJ (https://imagej.nih.gov/ij/) was used to quantify the protein levels by measuring the relative density of bands.

### Statistical analysis

All results were confirmed by at least two independent experiments. The sample size (*n*) represents the number of animals analyzed per group. Animals (biological replicates) were allocated into control or experimental groups on the basis of genotype and/or type of treatment. To avoid bias, masking was used during data analysis. Data were analyzed using Graphpad Prism 8.0. Relevant information for each experiment including sample size, statistical tests, and reported *P* values are found in the legend corresponding to each figure. In all cases, *P* < 0.05 was considered significant, and error bars represent SD. *P < 0.05, **P < 0.01, ***P < 0.001, and ****P < 0.0001.

## Notes

View/request a protocol for this paper from *Bio-protocol*.
