# Supplementary material for: Follistatin promotes LIN28B-mediated supporting cell reprogramming and hair cell regeneration in the murine cochlea
Source: Sci Adv. 2022 Feb 11;8(6):eabj7651. doi: 10.1126/sciadv.abj7651 (PMC8836811; doi:10.1126/sciadv.abj7651)
Supplement: Supplementary file 1 — Figs. S1 to S10 Legends for tables S1 to S5 Tables S6 to S9 [file sciadv.abj7651_sm.pdf]

Science Advances
AAAS

Supplementary Materials for

Follistatin promotes LIN28B-mediated supporting cell reprogramming and hair cell regeneration in the murine cochlea

Xiao-Jun Li, Charles Morgan, Loyal A. Goff, Angelika Doetzlhofer*

*Corresponding author. Email: adoetzlhofer@jhmi.edu

Published 11 February 2022, *Sci. Adv.* **8**, eabj7651 (2022)
DOI: 10.1126/sciadv.abj7651

The PDF file includes:

Figs. S1 to S10
Legends for tables S1 to S5
Tables S6 to S9

Other Supplementary Material for this manuscript includes the following:

Tables S1 to S5

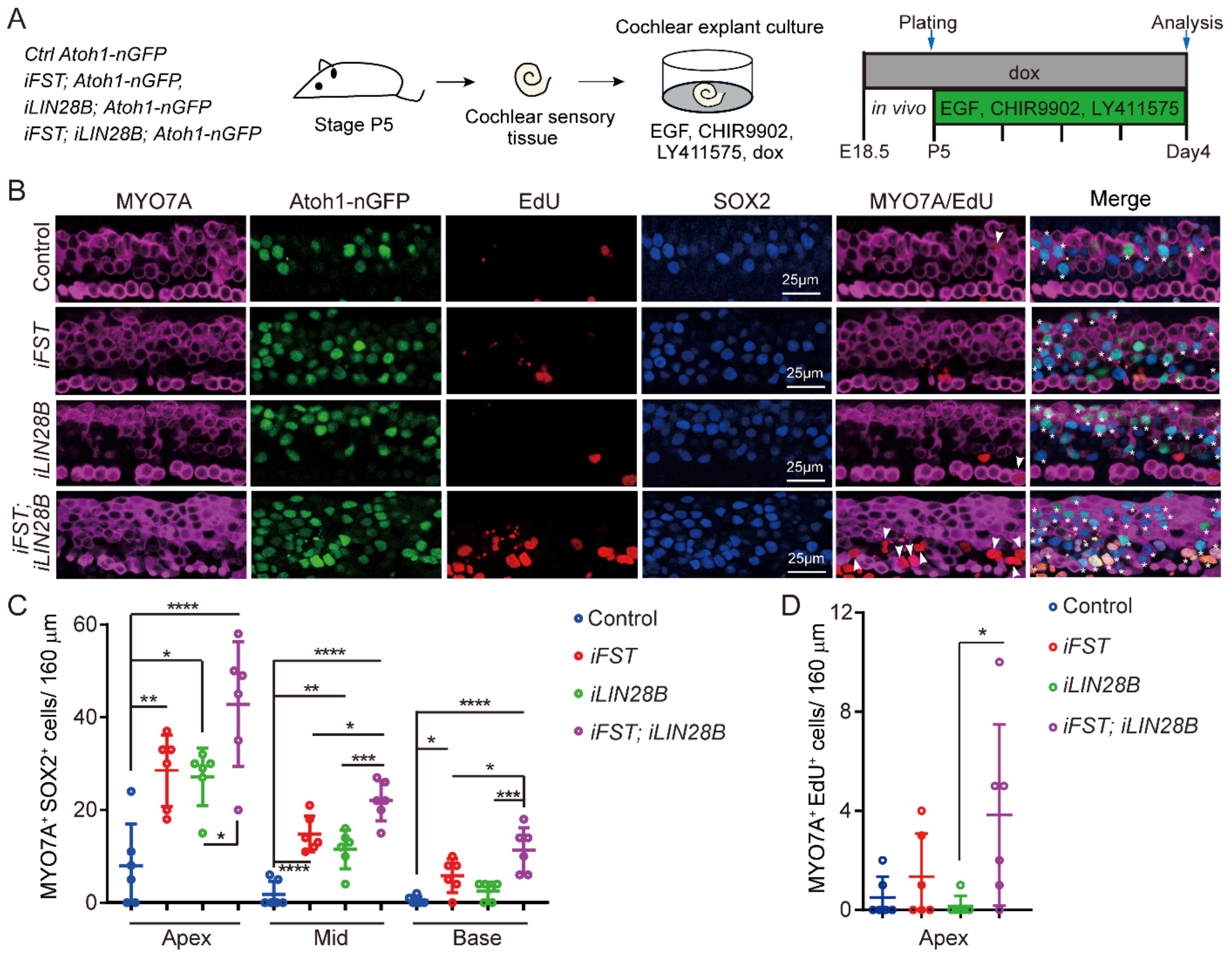

Fig. S1. FST and LIN28B co-activation enhances HC production in response to Wnt over-activation and Notch inhibition in stage P5 cochlear explants. (**A**) Experimental scheme. Pregnant dams received doxycycline (dox) containing feed starting at E18.5. Cochlear sensory tissue from *Atoh1-nGFP* control, *Atoh1-nGFP; iFST*, *Atoh1-nGFP; iLIN28B* and *Atoh1-nGFP; iFST; iLIN28B* transgenic mice was harvested at stage P5 and cultured in the presence of EGF (5 ng/mL), CHIR99021 (3 μM), LY411575 (5 μM), EdU (3 μM) and dox (10 μg/mL) for 4 days. Please note that Atoh1-nGFP reporter expression (green) is low in existing HCs but high in nascent HCs. (**B**) Confocal images of the HC layer of control (Ctrl, blue), FST (*iFST*, red), LIN28B (*iLIN28B*, green) and FST+LIN28B (*iFST*; *iLIN28B*, magenta) overexpressing cochlear explants. Shown are representative images of the sensory epithelia at the cochlear mid-apex. MYO7A immuno-staining (magenta) marks both existing and newly formed HCs. Co-labeling of MYO7A and SOX2 (blue) marks newly formed HCs (white asterisks). EdU (red) labels cells that proliferated in culture. White arrowheads mark HCs that formed by a mitotic mechanism. (**C**) Quantification of the number of new HCs ($MYO7A^{+}$ $SOX2^{+}$) that formed in the cochlear apex, mid and base. (**D**) Quantification of the number of HCs produced by a mitotic mechanism ($MYO7A^{+}$ EdU^{+}) in the cochlear apex. Graphed are average values for each animal and the mean ± SD, n=6 animals per group, two independent experiments, one-way ANOVA with Tukey's correction was used to calculate *P* values. *$*P < 0.05$, $**P < 0.01$, $***P < 0.001$, $****P < 0.0001$.*

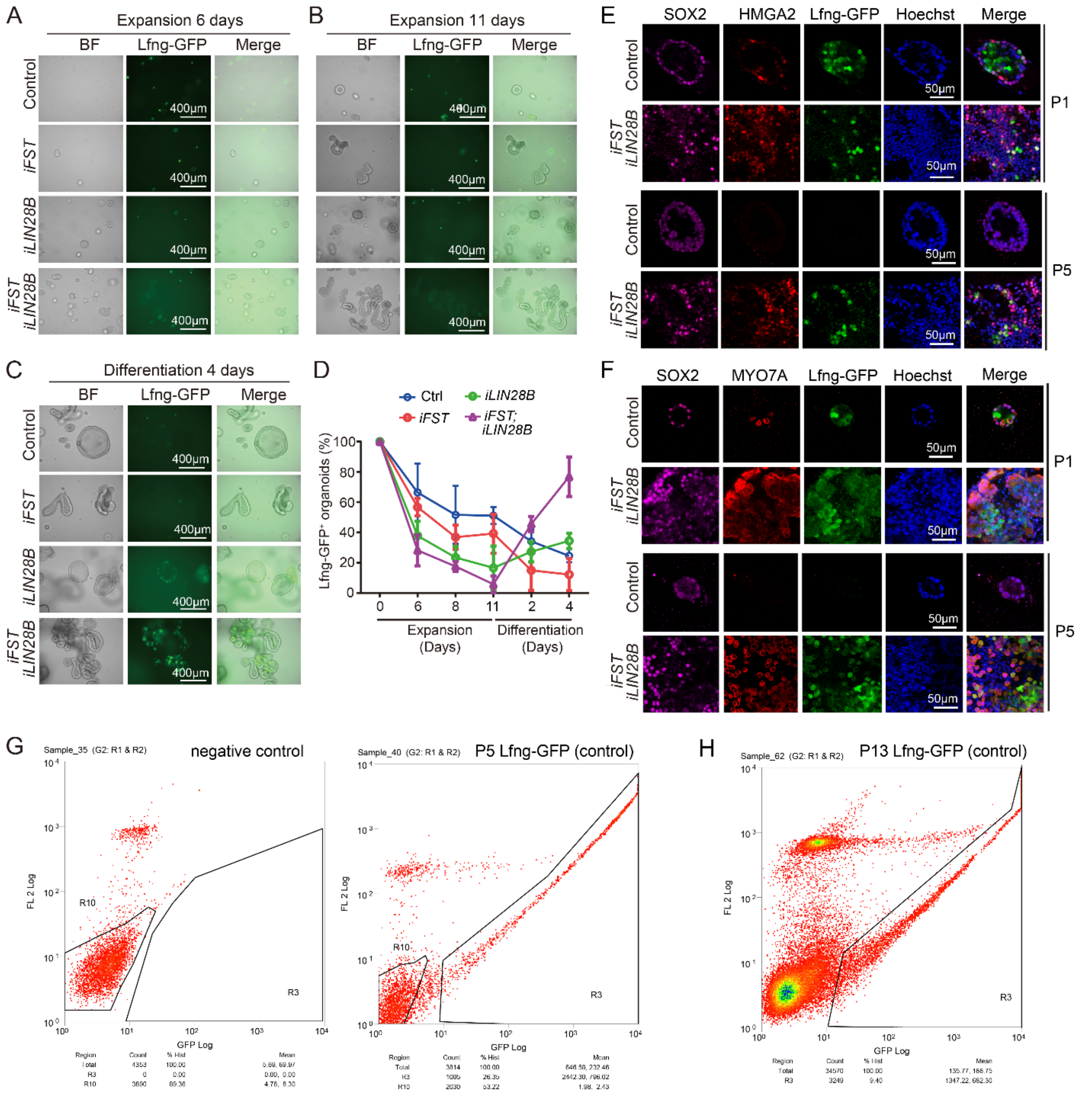

Fig. S2. FST and LIN28B co-activation promotes organoid expansion and HC formation in organoid cultures established with cochlear SCs. Organoid cultures were established with FACS purified Lfng-GFP$^+$ cochlear SCs from stage P5 control, *iFST*, *iLIN28B* and *iFST; iLIN28B* transgenic mice. **(A-C)** Bright field (BF) and green fluorescence (Lfng-GFP) images of control, FST (*iFST*), LIN28B (*iLIN28B*), and FST+LIN28B (*iFST; iLIN28B*) overexpressing organoid cultures after 6 (A) and 11 days of expansion (B) and 4 days of differentiation (C). **(D)** Percentage of Lfng-GFP$^+$ organoids after 6, 8, 11 days of expansion and 2 and 4 days of differentiation (n=3, for all the other groups). **(E-F)** Organoids established with Lfng-GFP$^+$ SCs isolated from control and *iFST; iLIN28B* transgenic mice stages P1 and P5 were expanded for 11 days and after 4 days of differentiation immuno-stained for SOX2 (magenta) and HMGA2 (red) (E) or SOX2 (magenta) and MYO7A (red) (F). Please note that some newly formed HCs co-express Lfng-GFP (green).

(**G, H**) Representative FACS plots and applied gates for isolation of Lfng-GFP$^+$ cells. Cell were stained with propidium iodine (red) to visualize dead cells. Lfng-GFP$^+$ cells were collected using gate R3. (**G**) FACS plots of non-transgenic (negative control) and Lfng-GFP transgenic cochlear epithelial cells stage P5. (**H**) FACS plot of Lfng-GFP transgenic cochlear cells stage P13.

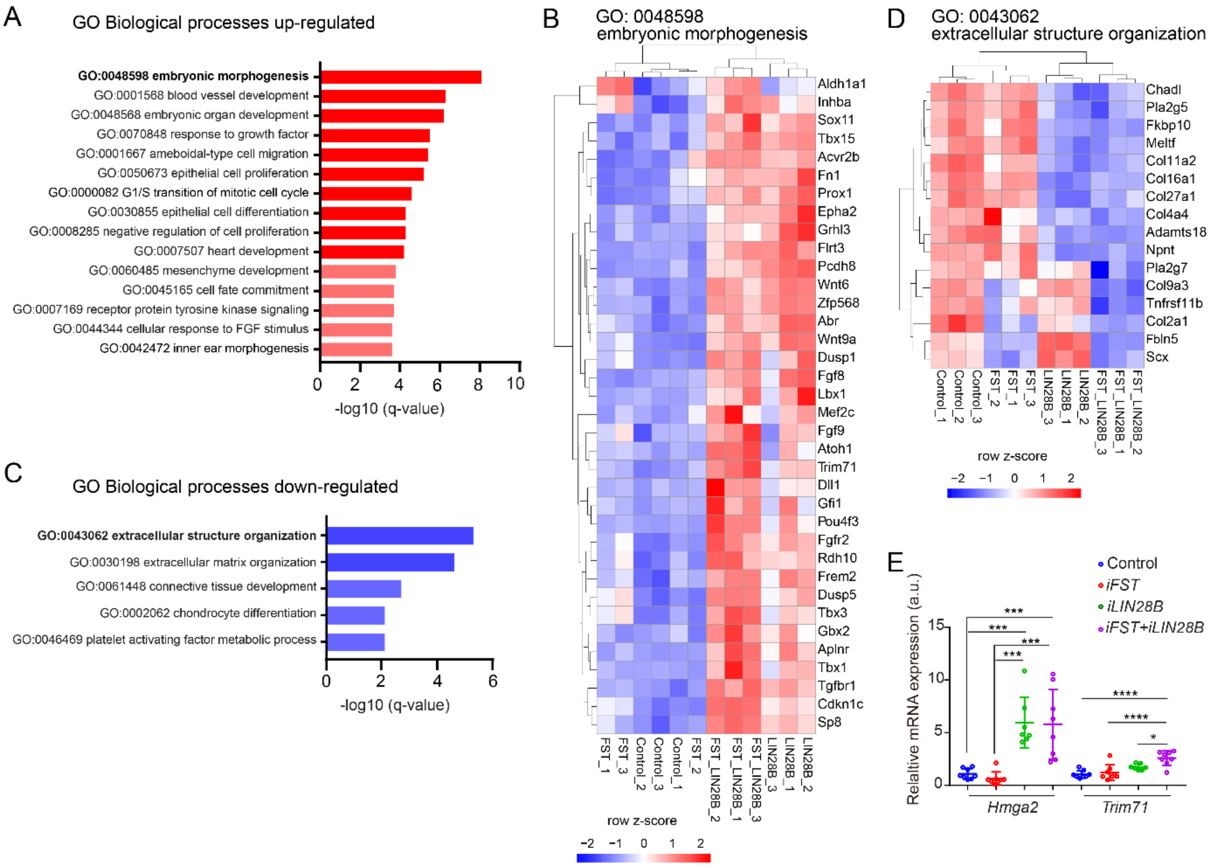

Fig. S3. FST and LIN28B co-activation differentially regulates genes associated with embryogenic morphogenesis and connective tissue organization. RNA-sequencing was used to analyze gene expression in P5 control, LIN28B, FST and FST+LIN28B overexpressing organoids after 7 days of expansion. **(A-D)** To identify biological processes regulated by co-activation of FST and LIN28B, gene ontology (GO) enrichment analysis was performed on significantly upregulated or down-regulated genes (FST+LIN28B vs. control). (**A**) Biological processes associated with upregulated genes ranked by adjusted p-value (q-value). Shown are the top 15. (**B**) Heat map of row z-scores computed for genes function in embryonic morphogenesis (top scoring biological process for upregulated genes). Each column in the heat map represents an individual sample. Row z-score ranging from blue to red (down to upregulated). (**C**) Biological processes associated with downregulated genes ranked by adjusted p-value (q-value). (**D**) Heat map of row z-scores computed for genes function in extracellular structure organization (top scoring biological process for downregulated genes). Each column in the heat map represents an individual sample. Row z-score ranging from blue to red (down to upregulated). **(E**) RT-PCR of pro-sensory-specific genes (*Hmga2* and *Trim71*) shows differential expression in control, *iFST*, *iLIN28B* and *iFST; iLIN28B* organoids after 7 days of expansion (n=7). Individual data points in (E) represent the average values per animal. n=animals analyzed per group. One-way ANOVA with Tukey's correction was used to calculate *P* values. $*P < 0.05$, $**P < 0.01$, $***P < 0.001$, $****P < 0.0001$.

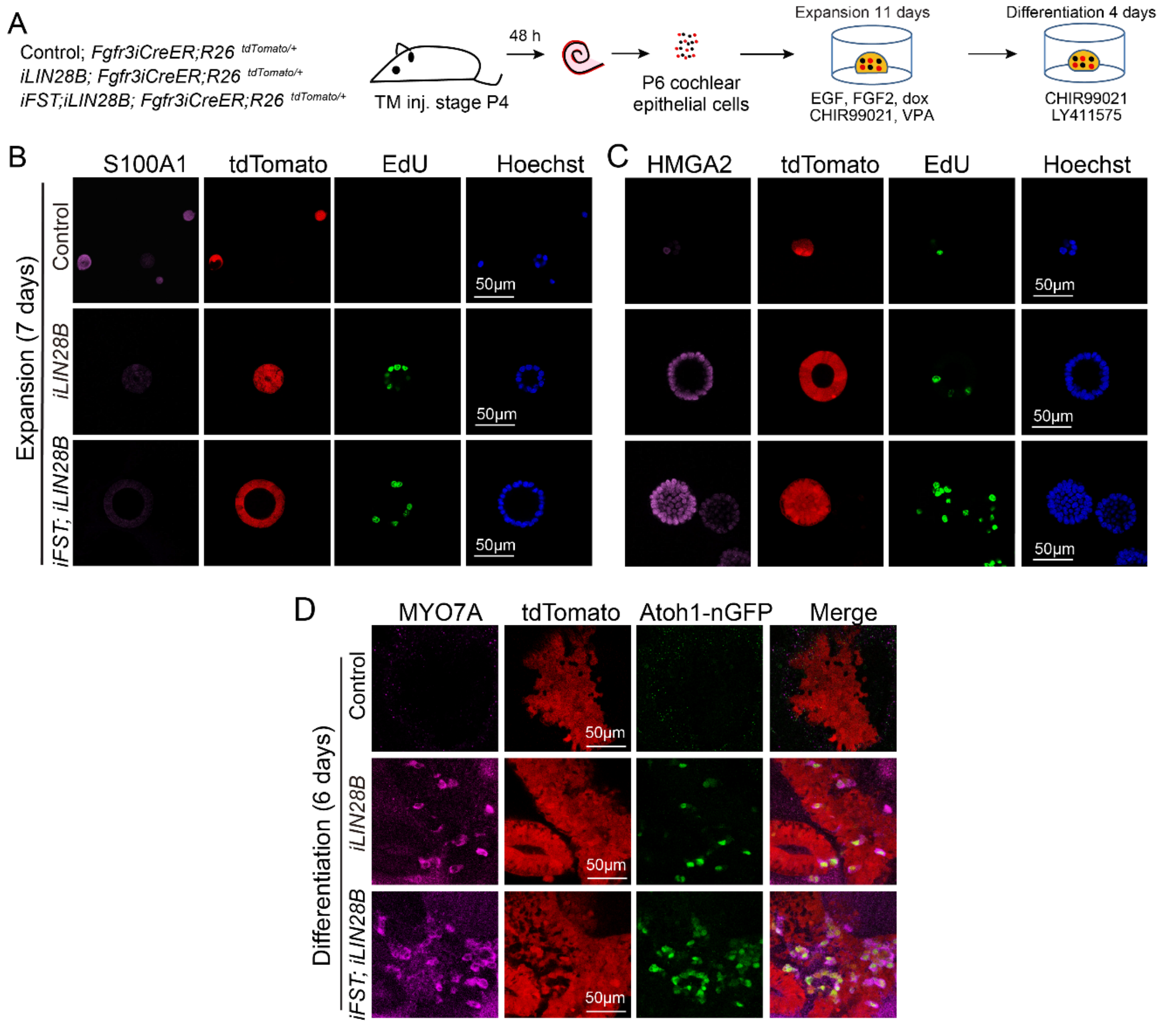

Fig. S4. Cochlear SCs dedifferentiate and produce HCs in organoid culture in response to LIN28B or FST and LIN28B overexpression. (A) Experimental scheme. Cochlear organoid cultures were established with cochlear sensory epithelial cells from stage P6 *iLIN28B* and *iFST; iLIN28B* transgenic mice and their control littermates. All mice also carried *Fgfr3iCreER; R26*^{tdTomato} transgenes to allow for lineage tracing. To permanently induce tdTomato expression in Deiter's cells and pillar cells, mice were injected with 4-OH-tamoxifen (TM) at stage P4. **(A-D)** High-power images of control, LIN28B (*iLIN28B*) and FST+LIN28B (*iFST; iLIN28B*) overexpressing organoid cultures after 7 days of expansion. EdU was added to expansion medium 1 hour before organoid harvest. EdU (green) labels cells undergoing DNA replication. **(B)** S100A1 (magenta) is downregulated in tdTomato^{+} Deiter's cells and pillar cells in response to LIN28B or FST+ LIN28B overexpression. (**C**) HMGA2 (magenta) is upregulated in tdTomato^{+} Deiter's cells and pillar cells in response to LIN28B or FST+LIN28B overexpression. (**D**) Confocal images of control, LIN28B (*iLIN28B*) and FST+LIN28B (*iFST; iLIN28B*) overexpressing organoids after 6 days of differentiation. Atoh1-nGFP (green) and MYO7A (magenta) labels new HCs, tdTomato (red) expression labels former Deiter's cells and pillar cells.

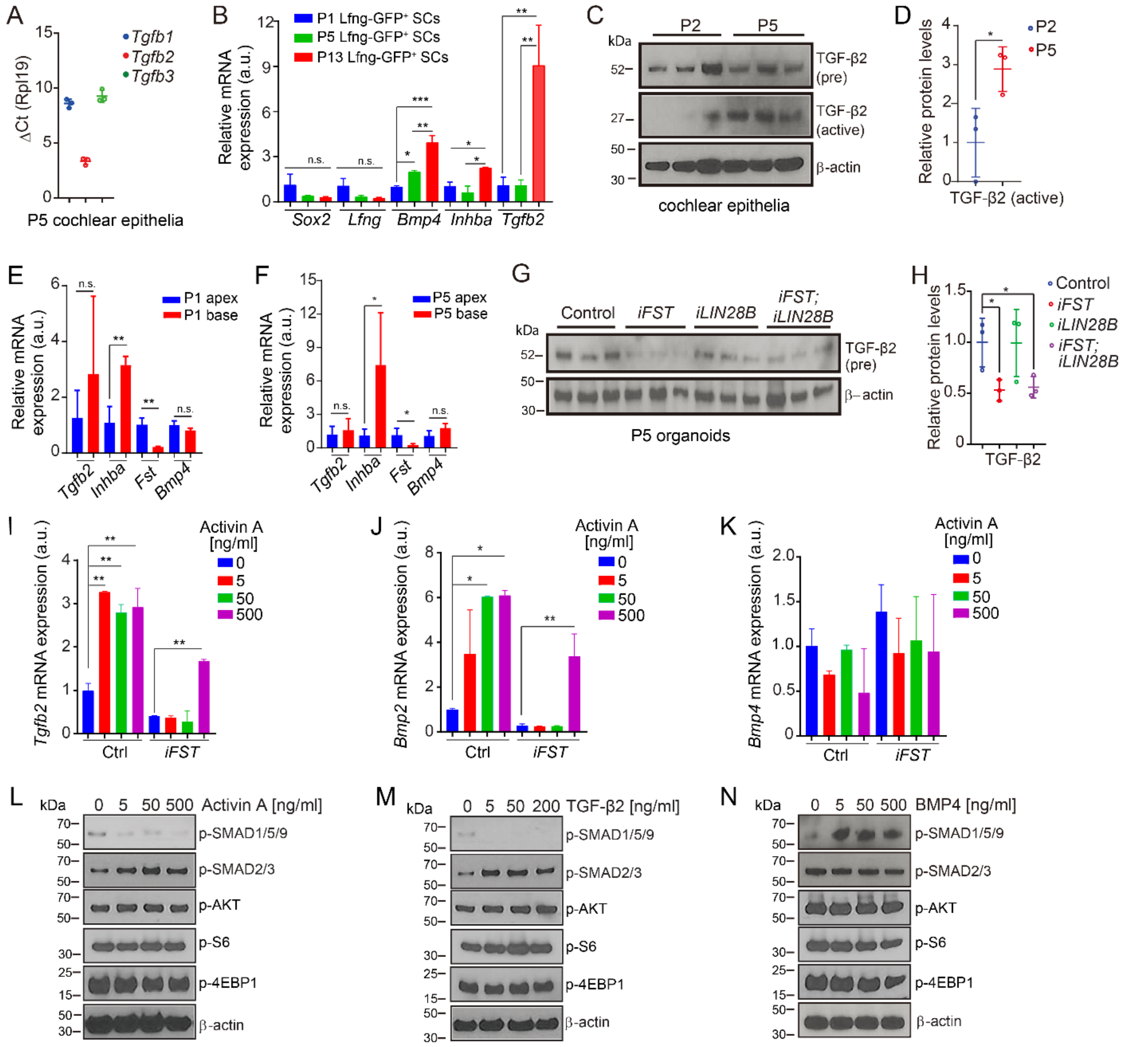

Fig. S5. FST represses Activin A-induced upregulation of *Tgfb2* and *Bmp2* expression. (**A**) RT-PCR was used to analyze *Tgfb1* (blue), *Tgfb2* (red) *and Tgfb3* (green) mRNA expression in cochlear sensory epithelia acutely isolated from P5 WT mice. Plotted are the differences in cycle threshold (ΔCT) compared to the reference gene *Rpl19* (n=3 animals per group). (**B**) RT-PCR was used to analyze *Sox2*, *Lfng*, *Bmp4*, *Inhba*, *Tgfb2* mRNA expression in Lfng-GFP$^+$ cochlear SCs that were FACS purified from P1 (blue), P5 (green) and P13 (red) Lfng-GFP transgenic mice (n=3 animals per group). (**C**) Immunoblots. TGF-β2 and β-actin protein levels were analyzed in protein lysates from acutely isolated cochlear sensory epithelia from P2 and P5 WT mice. (**D**) Normalized TGF-β2 protein level (active form) in B (n=3, from one representative experiment, two independent experiments). **(E-F)** Sensory epithelia from the apical and basal half of cochleae were isolated from P1 (E) and P5 (F) WT mice. RT-qPCR was used to compare *Tgfb2*, *Inhba*, *Fst* and *Bmp4* mRNA expression between apex (blue bars) and base (red bars) (n=3 animals per group) (**G**) Immunoblots of TGF-β2 and β-actin using protein lysates from organoids after 7 days of expansion. (**H**) Normalized TGF-β2 protein level shown in (**G**). (**I-K**) RT-qPCR analyzing *Tgfb2*,

Bmp2 and *Bmp4* after 8 days of expansion, Activin A was treated for 4 days from 4 days of expansion. (**L-N**) Organoid cultures established from stage P2 WT animals, cultured for 7 days in expansion media, were treated overnight w/o Activin A (**L**), TGF-β2 (**M**), BMP4 (**N**) after which organoids were harvested and protein levels of p-SMAD1/5/9, p-SMAD2/3, p-AKT, p-S6, p-4EBP1, and β-actin were analyzed using immunoblots. One-way ANOVA with Tukey's correction was used to calculate *P* values. $^{*}P < 0.05$, $^{**}P < 0.01$, $^{***}P < 0.001$.

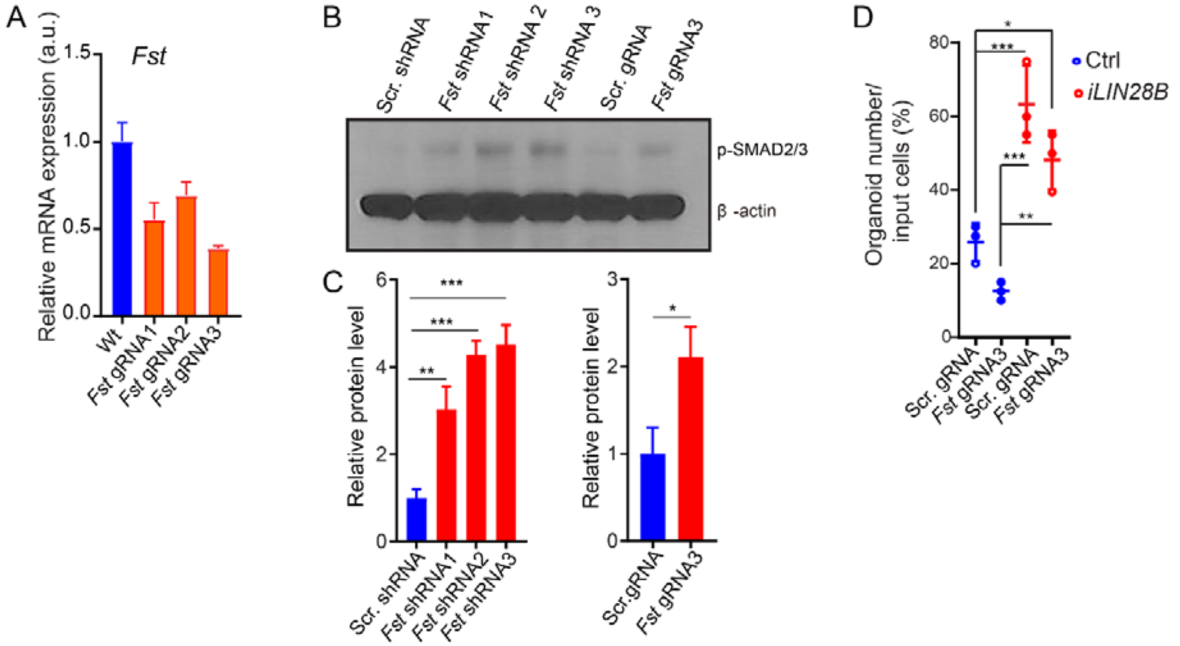

Fig. S6. (**A-C**) P19 cells were infected with lentivirus expressing Cas9 and guide *Fst*-specific guide (g) RNA constructs or *Fst*-specific shRNA constructs. As negative control, scramble (scr.) gRNA or scr. shRNA was used. Uninfected control and lentivirus-infected cells were harvested at 48 hours post infection and processed for RNA (A) or protein extraction (B). (**A**) RT-qPCR of endogenous *mouse Fst* mRNA expression following infection with *Fst*-specific guide (g) RNA constructs. (**B**) p-SMAD2/3 levels following infection with *scr.-shRNA*, *Fst-shRNA1*, *Fst-shRNA2*, *Fst-shRNA3*, *Scr.-gRNA* and *Fst-gRNA3* lentivirus. Expression of β-actin was used to normalize protein levels. (**C**) Quantification of p-SMAD2/3 protein levels in (B). Two-tailed, unpaired *t* test was used to calculate *P* values (n=3 biological replicates). (**D**) Organoid forming efficiency in stage P5 LIN28B overexpressing (*iLIN28B*) and control (ctrl) organoids infected with *Scr. gRNA* and *Fst-gRNA3* lentivirus at 11 days of expansion (n=3 mice per group, two independent experiment). Two-way ANOVA with Tukey's correction was used to calculate *P* values. $*P < 0.05$, $**P < 0.01$, $***P < 0.001$.

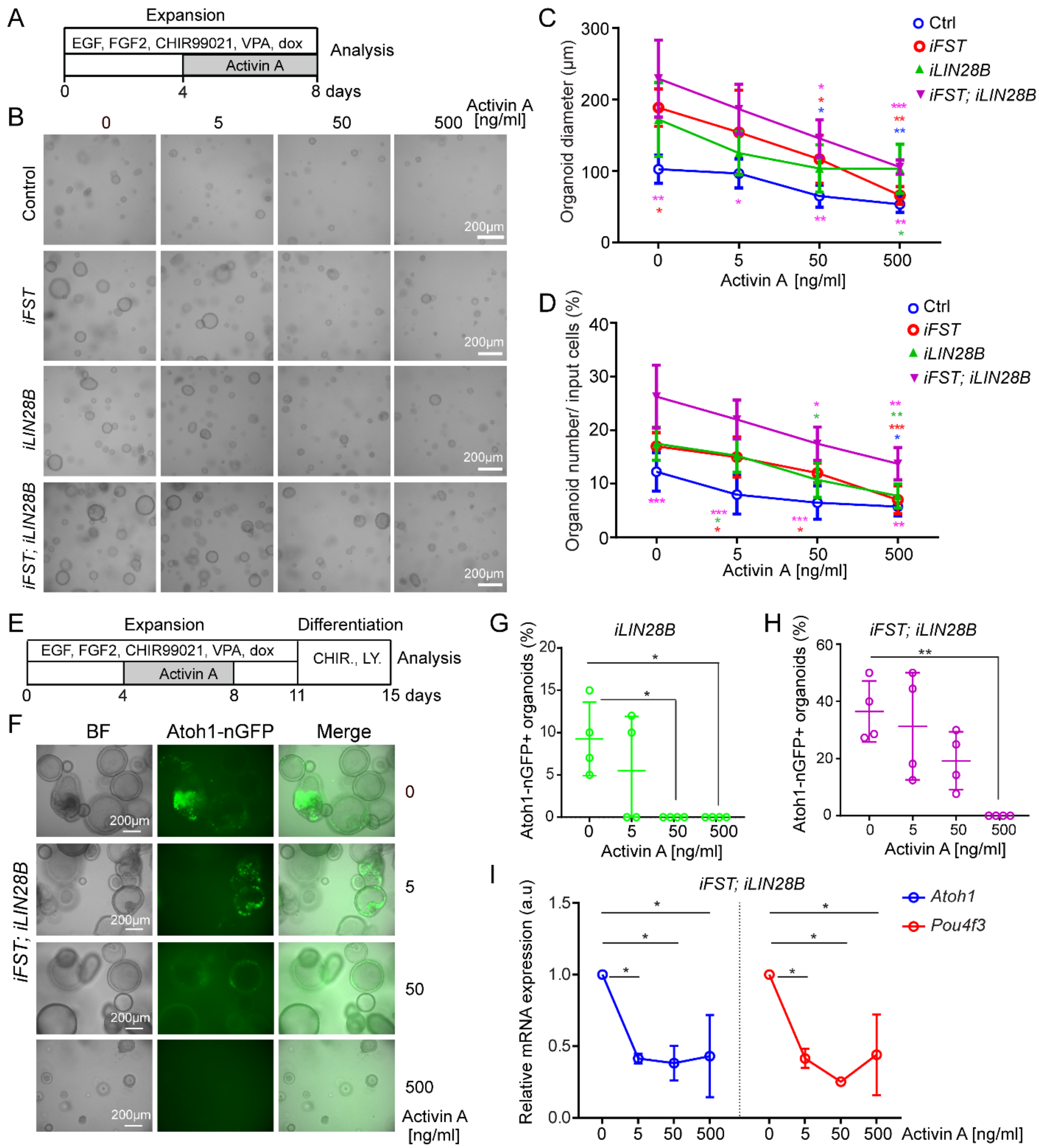

Fig. S7. Activin A inhibits HC formation in cochlear organoids. Organoid cultures were established with cochlear sensory epithelial cells from stage P5 control, *iFST*, *iLIN28B* and *iFST; iLIN28B* transgenic mice. To reactivate FST and or LIN28B expression expansion media contained doxycycline (dox). To be able to detect nascent HCs, mice were also transgenic for *Atoh1-nGFP*. **Exogenous Activin A inhibits organoid formation and growth.** **(A)** Experimental scheme. **(B)** Low-power BF images of Activin A treated and untreated organoids after 8 days of expansion. **(C)** Organoid diameter in (B) (n=4, from one representative experiment, three independent experiments). **(D)** Organoid forming efficiency in (B) (n=4, from one representative experiment, three independent experiments). **Activin A inhibits HC production in LIN28B or**

LIN28B+FST overexpressing organoids. (E) Experimental scheme. **(F)** High-power BF and green fluorescent (Atoh1-nGFP) images of FST+LIN28B overexpressing organoids after 4 days of differentiation. **(G)** Percentage of Atoh1-nGFP+ organoids in Activin A-treated and untreated LIN28B overexpressing organoid cultures (n= 4, from one representative experiment, three independent experiments). **(H)** Percentage of Atoh1-nGFP+ organoids in Activin A treated and untreated FST+LIN28B overexpressing organoid cultures shown in (F) (n= 3 for untreated control, n=4 Activin A treated group, from one representative experiment, three independent experiments). **(I)** RT-PCR analyzing *Atoh1* (blue) and *Pou4f3* (red) mRNA induction in Activin A treated and untreated FST+LIN28B overexpressing organoids in (F) (n= 3, from one representative experiment, three independent experiments). One-way ANOVA with Tukey's correction was used to calculate P values in (C, D, G, H). Two-tailed, unpaired t test was used to calculate P values in (I). $^{*}P < 0.05$, $^{**}P < 0.01$, $^{***}P < 0.001$.

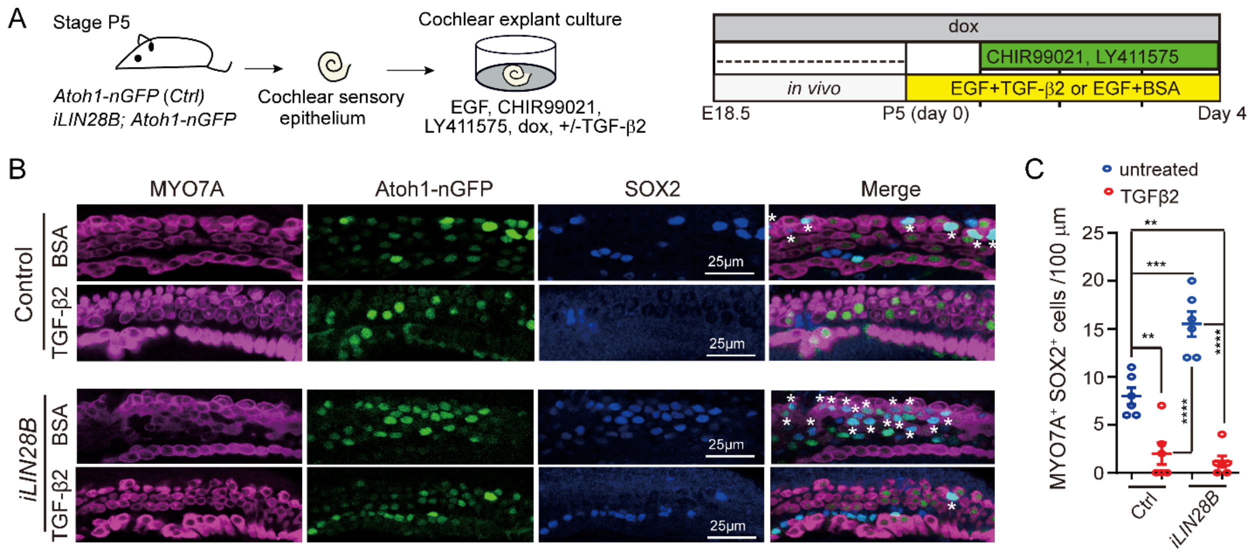

Fig. S8. TGF-β2 inhibits HC production in response to Notch inhibition and Wnt activation in stage P5 cochlear explants. (**A**) Experimental strategy. Pregnant dams were fed doxycycline (dox) containing feed starting at E18.5. Cochlear tissue from *Atoh1-nGFP* control and *Atoh1-nGFP; iLIN28B* transgenic mice was harvested at stage P5 and cultured in the presence of dox (10 µg/mL), EGF (5 ng/mL) and TGF-β2 (5 ng/mL) or BSA (vehicle control) for 4 days. Wnt activator CHIR99021 (3µM) and Notch inhibitor LY411575 (5 µM) were added one day after plating. Please note that Atoh1-nGFP reporter expression (green) is low in pre-existing HCs but high in nascent HCs. (**B**) High-power single plane confocal images of the HC layer of control (Ctrl, blue) and LIN28B (*iLIN28B*, red) overexpressing cochlear explants. Shown are representative images of the cochlear mid-apex. MYO7A staining (magenta) marks both pre-existing and newly formed HCs. Co-labeling of MYO7A with SOX2 (blue) marks newly formed HCs (white asterisks). (**C**) Quantification of newly formed HCs (MYO7A$^+$ SOX2$^+$) in (B). Graphed are average values for each animal and the mean ± SD, n=6 animals per group, two independent experiments, two-way ANOVA with Tukey's correction was used to calculate *P* values. **P < 0.05, **P < 0.01, ***P < 0.001, ****P < 0.0001.*

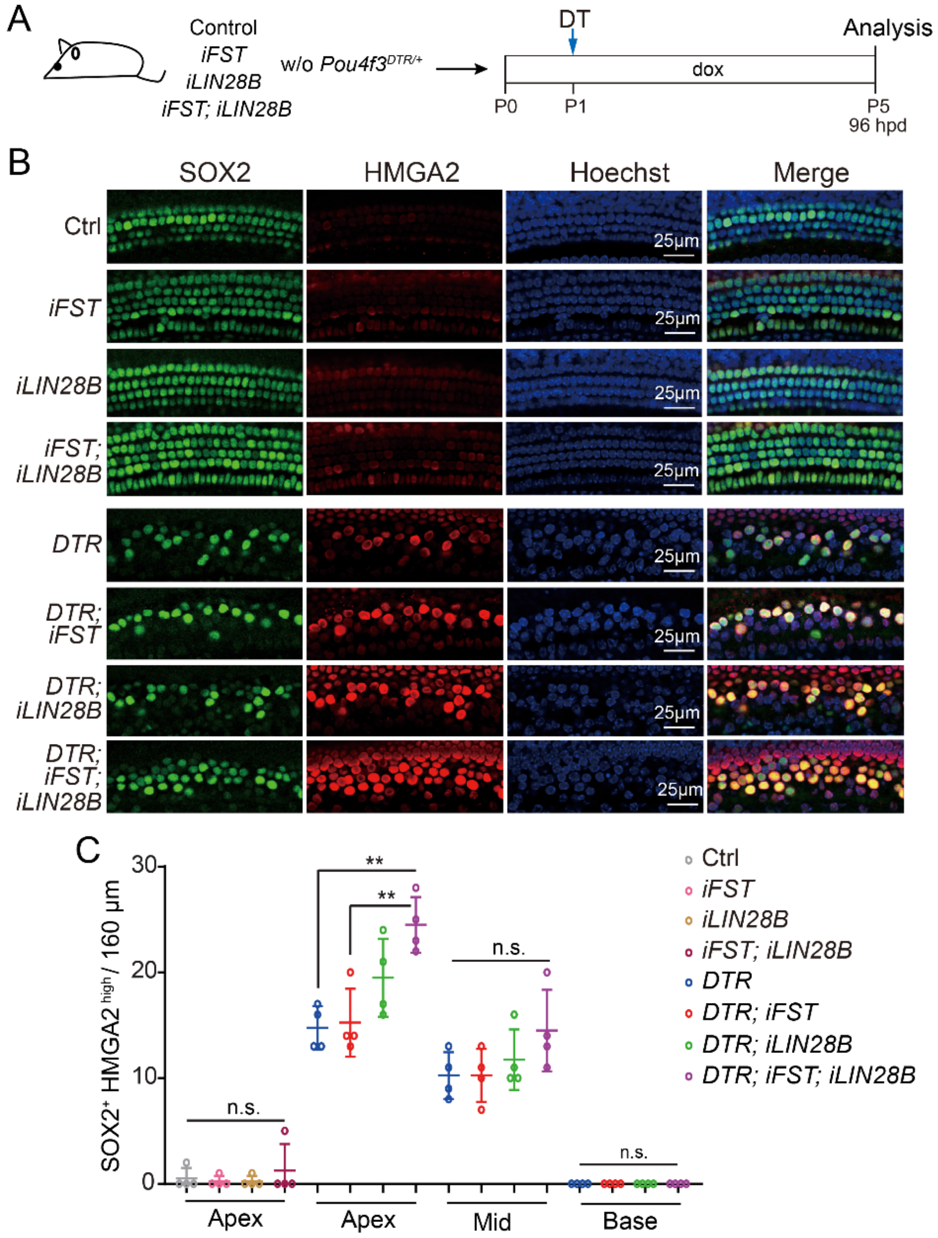

Fig. S9. Co-activation of FST and LIN28B enhances HMGA2 induction in SCs following HC damage. (**A**) Experimental scheme. Control (Ctrl), *iFST*, *iLIN28B* and *iFST; iLIN28B* transgenic mice w/o *Pou4f3*$^{DTR/+}$ transgene received dox starting at P0 and a single injection of diphtheria toxin (DT) at stage P1. (**B**) High-power single plane confocal images showing SOX2 (green) and HMGA2 (red) expression in intact control (Ctrl), FST (*iFST*), LIN28B (*iLIN28B*) and FST+LIN28B (*iFST; iLIN28B*) overexpressing cochlear sensory epithelia and HC damaged control (DTR), FST (*DTR; iFST*), LIN28B (*DTR; iLIN28B*) and FST+LIN28B (*DTR*; *iFST; iLIN28B*) overexpressing cochlear sensory epithelia 96 hours post DT injection (hpd). (**C**) Quantification of SOX2^{+} HMGA2^{high} cells in acutely isolated cochlear sensory tissue 96 hpd. Graphed are average values for each animal and the mean ± SD, n=3 in undamaged group and n=4 in HC damaged group, one-way ANOVA with Tukey's correction was used to calculate *P* values in (C). **P < 0.05, **P < 0.01*.

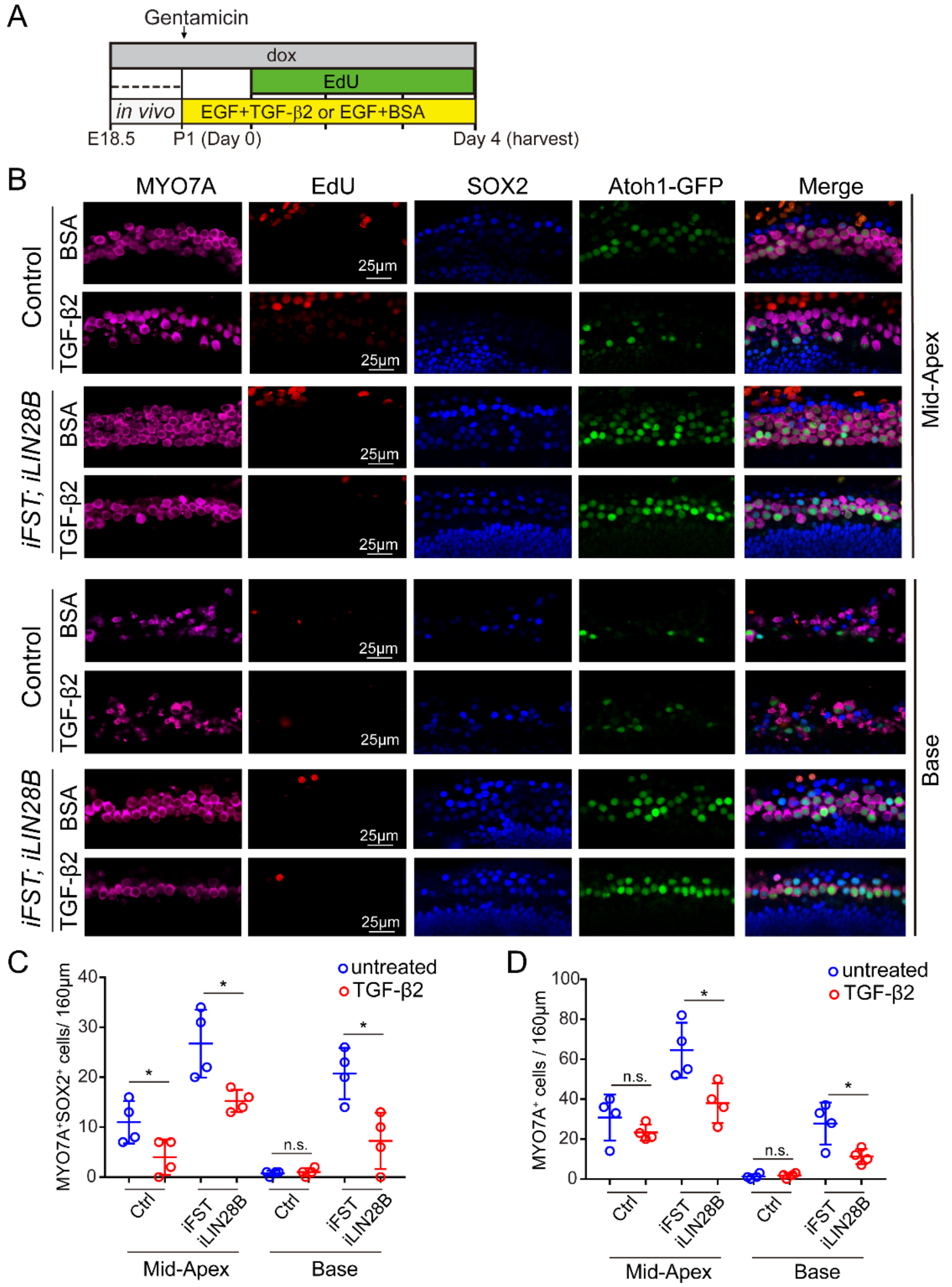

Fig. S10. TGF-β2 inhibits spontaneous HC regeneration in stage P1 cochlear explants. (**A**) Experimental strategy. Pregnant dams received doxycycline (dox) containing feed starting at E18.5. The cochlear sensory tissue from *Atoh1-nGFP* control and *Atoh1-nGFP; iFST; iLIN28B* transgenic mice was harvested at stage P1 and cultured in the presence of dox (10 µg/mL), EGF (5 ng/mL) and TGF-β2 (5 ng/mL) or BSA (vehicle control TGF-β2) for 4 days. To ablate HCs, cochlear explants received gentamicin (100 µg/mL) at plating. After 20 h the gentamicin-containing media was replaced and EdU was added to mark dividing cells. (**B**) High-power single plane confocal images of control and FST+LIN28B (*iFST; iLIN28B*, red) overexpressing cochlear sensory epithelia stained for MYO7A (magenta), EdU (red) and SOX2 (blue). MYO7A and Atoh1-

nGFP (green) marks new and existing HCs. MYO7A and SOX2 mark new HCs. (**C**) Quantification of newly formed HCs ($MYO7A^{+}SOX2^{+}$) in (B). **(D)** Quantification of the total number of HCs ($MYO7A^{+}$) in (B). Graphed are average values for each animal and the mean ± SD, n=4 animals per group, two independent experiments, Two-tailed, unpaired *t* tests was used to calculate *P* values. $^{*}P < 0.05$.

Tables S1-S5 are provided as separate files in spreadsheet format

Table S1 Wald test Ctrl vs FST+LIN28B

Table S2 Wald test Ctrl vs LIN28B

Table S3 Wald test Ctrl vs FST

Table S4 GO analysis Ctrl vs FST+LIN28B upregulated

Table S5 GO analysis Ctrl vs FST+LIN28B downregulated

Mouse line	Genotyping primers	Product size
Atoh1-GFP	EGFP1: CGA AGG CTA CGT CCA GGA GCG CAC<br>EGFP2: GCA CGG GGC CGT CGC CGA TGG GGG TGT	TG= 300bp
R26 ^{rtTA*M2}	MTR: GCG AAG AGT TTG TCC TCA ACC<br>F: AAA GTC GCT CTG AGT TGT TAT<br>WTR: GGA GCG GGA GAA ATG GAT ATG	WT=650bp<br>MT=340bp
Col1A1 (*LIN28B*)	ColA: GCA CAG CAT TGC GGA CAT GC<br>ColB: CCC TCC ATG TGT GAC CAA GG<br>ColC: GCA GAA GCG CGG CCG TCT GG	WT=300bp<br>TG=450bp
iFST	YA88: TTGCCTCCTGCTGCTGCTGC<br>YA123: TTTTTCCCAGGTCCACAGTCCACG	TG=247bp
Lfng-GFP	EGFP1: CGA AGG CTA CGT CCA GGA GCG CAC<br>EGFP2: GCA CGG GGC CGT CGC CGA TGG GGG TGT	TG= 300bp
Fgfr3iCreER	iCre-F: GAG GAC TAC CTC CTG TAC C<br>iCre-R: TGC CCA GAG TCA TCC TTG G	TG:600bp
R26 ^{tdTomato}	WT-F: AAG GGA GCT GCA GTG GAG TA<br>WT-R: CCG AAA ATC TGT GGG AAG TC<br>MT--F: CTG TTC CTG TAC GGC ATG G<br>MT-R: GGA ATT AAA GCA GCG TAT CC	WT:300bp<br>MT:200bp
Pou4f3 $^{DTR/+}$	Common: AAG AAG CAG GTG GGG GAG AG<br>Wild type Reverse: ATT GTT CTG GGC GAC ATG A<br>Mutant Reverse: CAG AAA GAG CTT CAG CAC CAC	Mutant: 290bp<br>WT:351bp

Table S6. **List of genotyping primers.**

Gene	Forward Primer	Reveres Primer
Atoh1	ATG CAC GGG CTG AAC CA	TCG TTG TTG AAG GAC GGG ATA
Gfi1	AGGAACGCAGCTTTGACTGT	TGAGATCCACCTTCCTCTGG
Myo7a	CCC CCT CTG AGA AGT TCG TTA	TGT GTC CGA GTT CCG TTG AC
Pou4f3	GCA CCA TCT GCA GGT TCG A	CCG GCT TGA GAG CGA TCA T
Fst	GAA AAC CTA CCG CAA CGA ATG	TCC GGC TGC TCT TTG CAT
Hmga2	CAG AAG AAA GCA GAG ACC ATT	TTG TTG TGG CCA TTT CCT AGG T
Trim71	ATC GGG AGT GTG AGC TGT TG	GGC GTG AAC ATA ATG CGG TC
Lin28a	TCC AAA GGA GAC AGG TGC TAC	TTG CAT TCC TTG GCA TGA TG
Lin28b	CAT GGC ACT GGC CAC TGT AA	ATC ATG GAG ATG AAT CCG AAT
Sox2	CCA GCG CAT GGA CAG CTA	GCT GCT CCT GCA TCA TGC T
Lfng	ACT GCA CCA TTG GCT ACA TTG	GGC CGC TCC GGA TGA
Bmp2	CTG TCC CCA GTG ACG AGT TTC	CCT GTA TCT GTT CCC GGA AGA
Bmp4	ACG TAG TCC CAA GCA TCA CC	ACT AGG GTC TGC ACA ATG GC
Inhba	GGG TAA AGT GGG GGA GAA CG	ACT TCT GCA CGC TCC ACT AC
Tgfb1	GAG CCC GAA GCG GAC TAC TAT	CAG CCA CTG CCG TAC AAC TCC
Tgfb2	GAG CGG AGC GAC GAG GAG T	ACA GCA GGG GCA GTG TAA ACT
Tgfb3	AGA GGG CCC TGG ACA CCA A	CTG GCC TCA GCT GCA CTT ACA C
Mmp14	CTG CCA TTG CCG CCA TGC AAA	TGG CGT GGC ACT CTC CCA TAC T
Scx	CTT CAC TGC GCT GCG CAC ACT	GCT CTC CGT GAC TCT TCA GTG
Twist1	GGA CAA GCT GAG CAA GAT TCA	CGG AGA AGG CGT AGC TGA G
Zeb1	GCA GTT ACA CCT TTG CAT ACA	GTC TTT GCT CTC TTC CTG ACT
Zeb2	TGA CAT GAC AGA CTC CGA TTC	TGC ATA CAT GCC ACT TTC TGT
CFP	CCA TGC CCG AAG GCT ACG	CTG CCG TCC TCG ATG TTG TG
RFP	GAAGCACCCCGCCGACATCC	GGC AGC TGC ACG GGC TTC TTG
ZsGreen	CCC TTC GCC GAG GAC ATC T	GGG GAA GTT CAC GCC GTA GA
Rpl19	GGT CTG GTT GGA TCC CAA	TGC CCG GGA ATG GAC AGT CA

Table S7. List of qPCR primers.

**Reagent type**	**Designation**	**Source**	**Identifiers**	**Additional information**
antibody	myosin VIIa<br>rabbit polyclonal	Proteus Biosciences	Cat.# 25-6790	1:500 dilution
antibody	S100A1<br>rabbit polyclonal	Abcam	Cat.# ab11428	1:500 dilution
antibody	SOX2<br>goat polyclonal	Santa Cruz	Cat.# sc-17320	1:500 dilution
antibody	HMGA2<br>rabbit monoclonal	Cell Signaling	Cat.# 8179	1:5000 dilution
antibody	donkey anti-rabbit<br>IgG (H+L) Alexa Fluor 546	ThermoFisher	Cat.# A10040	1:1000 dilution
antibody	donkey anti-rabbit IgG (H+L) Alexa Fluor 647	ThermoFisher	Cat.# A-31573	1:1000 dilution
antibody	donkey anti-mouse IgG (H+L) Alexa Fluor 546	ThermoFisher	Cat.# A-10036	1:1000 dilution
antibody	donkey anti-goat IgG (H+L) Alexa Fluor 488	ThermoFisher	Cat.# A-11055	1:1000 dilution
antibody	donkey anti-goat IgG (H+L) Alexa Fluor 546	ThermoFisher	Cat.# A-11056	1:1000 dilution
antibody	Biotin-SP (long spacer) AffiniPure Donkey Anti-Goat IgG (H+L)	Jackson Immuno Research Lab	Cat.#705-065-147	1:200 dilution
biotin-binding protein	Streptavidin, Alexa Fluor™ 405 conjugate	Life Technologies	Cat. #S32351	1:200 dilution
nuclear stain	Hoechst 33258 solution	Sigma-Aldrich	Cat.# 94403	1:3000 dilution

Table S8. List of antibodies and stains used for immunostaining

Reagent type	Designation	Source	Identifiers	Additional information
antibody	p-Akt<br>rabbit monoclonal	Cell Signaling	Cat.# 4060	1:1000 dilution
antibody	p-4E-BP1rabbit monoclonal	Cell Signaling	Cat.# 2855	1:2000 dilution
antibody	p-S6<br>rabbit monoclonal	Cell Signaling	Cat.# 5364	1:1000 dilution
antibody	β-actin<br>mouse monoclonal	Santa Cruz	Cat.# 47778	1:500 dilution
antibody	p-Smad2/3<br>rabbit monoclonal	Cell Signaling	Cat.# 8828	1:1000 dilution
antibody	p-Smad1/5/9<br>rabbit monoclonal	Cell Signaling	Cat.#13820	1:1000 dilution
antibody	Hmga2<br>rabbit monoclonal	Cell Signaling	Cat.# 8179	1:1000 dilution
antibody	S100A1<br>rabbit monoclonal	Abcam	Cat:#ab11428	1:1000 dilution
antibody	TGF-β2 mouse monoclonal	Santa Cruz	Cat:# SC-374659	1:500 dilution

Table S9. Antibodies for Immunoblotting.
